# DeepEPI: CNN-transformer-based model for extracting TF interactions through predicting enhancer-promoter interactions

**DOI:** 10.1093/bioadv/vbaf221

**Published:** 2025-09-17

**Authors:** Seyedeh Fatemeh Tabatabaei, Saeedeh Akbari Roknabadi, Somayyeh Koohi

**Affiliations:** Department of Computer Engineering, Sharif University of Technology, Tehran, 11155-9517, Iran; Division of Computational Science and Technology, KTH Royal Institute of Technology, Stockholm, 17121, Sweden; Department of Computer Engineering, Sharif University of Technology, Tehran, 11155-9517, Iran

## Abstract

**Motivation:**

We introduce DeepEPI, a deep learning framework for studying enhancer–promoter interactions (EPIs) directly from genomic sequences. By integrating convolutional neural networks (CNNs) with Transformer blocks, DeepEPI captures the complex regulatory interplay between enhancers and promoters, a key factor in gene expression and disease mechanisms. The model emphasizes interpretability and efficiency by employing embedding layers for OneHot encoding and multihead attention for detecting and analyzing transcription factor (TF) interactions. A DNA2Vec-based version of DeepEPI is also evaluated.

**Results:**

DeepEPI is assessed in two dimensions: comparison with existing models and analysis of encoding methods. Across six cell lines, DeepEPI consistently outperforms prior approaches. Compared to EPIVAN, it achieves a 2.4% gain in area under the precision-recall curve (AUPR) and maintains AUROC with DNA2Vec encoding, while with OneHot encoding it shows a 4% increase in AUPR and 1.9% in AUROC. Regarding encoding, DNA2Vec provides higher accuracy, but our OneHot-based embedding balances competitive performance with interpretability and reduced storage requirements. Beyond prediction, DeepEPI enhances biological insight by extracting meaningful TF–TF interactions from attention heads, effectively narrowing the search space for experimental validation. Validation analyses further support the biological relevance of these findings, underscoring DeepEPI’s value for advancing EPI research.

**Availability and implementation:**

The source code of DeepEPI is available at: https://github.com/nazanintbtb/DeepEPI.git.

## 1 Introduction

Promoters and enhancers play crucial roles in the intricate orchestration of gene expression within cells. While promoters determine the initiation site of transcription and regulate gene expression frequency, enhancers amplify the transcriptional activity of specific genes, often working in conjunction with promoters to fine-tune expression levels (Mora *et al.* 2016). Consequently, enhancer-promoter interactions (EPIs) are pivotal in shaping gene expression patterns and are closely linked to the onset of various human diseases (Williamson *et al*., 2011). Therefore, detecting EPIs is crucial in medicine and treatment, as their impact extends beyond human health. However, identifying these interactions is challenging due to the complexity of their modes of interaction; a single enhancer can influence one or multiple target promoters, and conversely, several enhancers may jointly regulate a single target promoter (van Arensbergen *et al.* 2014). Additionally, the spatial relationship between enhancers and promoters lacks fixed positions, presenting a challenge for traditional biological experimental studies. These methods are not only confronted by these natural challenges but are also inherently expensive. Nevertheless, advancements in high-throughput sequencing technologies such as high-throughput chromosome conformation capture (Hi-C) (Rao *et al.* 2014) and chromatin interaction analysis using paired-end tag sequencing (ChIA-PET) have facilitated the generation of vast amounts of data, paving the way for computational approaches to unravel the mysteries of EPIs (Heidari *et al.* 2014).

In recent years, machine learning-based computational methods have revolutionized the study of EPIs, offering efficient and accurate means to identify these interactions on a genomic scale. Notably, deep learning approaches have emerged as powerful tools for predicting EPIs solely from genomic sequences. However, other tools exist that rely on different data. For instance, TargetFinder (Whalen *et al.* 2016) is an EPIs prediction model trained with multiple genomic peak data, like DNase-seq, DNA methylation, transcription factor ChIP-seq, histone marks, CAGE, and gene expression data, as model features. Although these methods achieve slightly higher accuracy, a limitation of such methods is the requirement for relevant knowledge to select appropriate genomic features and the lack of available data.

Examples of solely sequence-based tools include the method by Yang *et al.* (2017), which introduced a predictive algorithm that utilizes word embedding from genomic sequences to enhance performance through a boosted tree ensemble model, demonstrating the effectiveness of sequential features in improving the predictive accuracy of EPIs. Similarly, Mao et al. (2017) proposed the EPIANN model, a neural network architecture incorporating attention mechanisms and location-based feature decoding to effectively identify EPIs, highlighting the importance of attentive feature extraction in predictive modeling. Some years later, Singh *et al.* (2019) introduced SPEID, a deep learning-based predictive model that integrates CNN with long short-term memory (LSTM) networks to leverage spatial and sequential information in genomic sequences for enhanced EPI prediction and extract motifs from filters of CNN by convert each kernel into a PFM matrix. Furthermore, Zhuang *et al.* (2019) simplified the SPEID model by employing CNN coupled with transfer learning, resulting in the creation of SIMCNN, a computational tool optimized for efficient prediction of EPIs across diverse genomic contexts. After that Hong *et al.* (2020) made a slight change in this structure and introduced EPIVAN, a deep learning-based predictive model that integrates CNN with gate recurrent unit (GRU) and attention mechanism to improve prediction accuracy in EPIs also they are using encoding approach name DNA2Vec, and EPI-Trans. Ahmed *et al.* (2024) introduced a transformer-based model, using DNA2Vec for encoding the sequences. This approach has shown a slight improvement in accuracy compared to previous models but lacks interpretability features. An important point about these methods is that only PEPWORD, TF-EPI, SPEID, and EPIANN support the identification of TFs, and among them, EPIANN is the only one that identifies TF interactions which is essential in fields such as drug development. Table 1 demonstrates summery of tools and their ability to identify TFs and TFs’ interactions.

**Table 1.** Summary of previous tools.

Tool name	Encoding	Architecture	Identify TFs	Identify TF interactions
PEPWORD (Yang *et al.* 2017)	Word embedding	Boosted tree	+	−
SPEID (Singh *et al.* 2019)	OneHot	CNN-LSTM	+	−
SIMCNN (Zhuang *et al.* 2019)	OneHot	CNN	−	−
EPIANN (Mao *et al.* 2017)	Word embedding	Attention mechanism	+	+
EPI-Trans (Ahmed *et al.* 2024)	DNA2Vec	CNN-transformer	−	−
EPIVAN (Hong *et al.* 2020)	DNA2Vec	CNN-GRU-attention	−	−

Despite significant progress in existing studies, some challenges persist. One major hurdle is the limitations of EPIs models in accurately identifying TFs and their interactions (Wang *et al.* 2022), as well as determining the specific interactions between motifs in enhancers and promoters. Additionally, the choice between OneHot encoding and word embedding for sequence encoding poses a challenge, with OneHot encoding requiring substantial storage and not satisfaction performance compared to the word embedding approach while it is a good choice for model interpretation. These remaining obstacles underscore the need for further exploration and refinement in the field of genomics research. To address these limitations, we propose DeepEPI, a novel deep learning model based on CNN and transformer blocks for predicting EPIs solely from genomic sequences. Our work offers three key contributions: (i) The utilization of an embedding layer for OneHot encoding to reduce storage requirements, (ii) The integration of a transformer block to enhance prediction accuracy by extracting more refined feature dependencies. (iii) The incorporation of a multihead attention layer for capturing motifs and TFs interactions. Performance evaluation conducted on six different cell lines illustrates that DeepEPI surpasses the performance of existing models. Our methodology proves advantageous for biological researchers and complements the existing techniques for computationally identifying EPIs.

## 2 Methods

### 2.1 Data

In our study, we utilize the EPI dataset obtained from TargetFinder (Whalen *et al.* 2016), which we mirror to rigorously assess and benchmark our model against existing methodologies. This dataset encompasses a diverse range of EPIs across six distinct human cell lines:

- GM12878: lymphoblastoid cells
- HUVEC: umbilical vein endothelial cells
- HeLa-S3: ectoderm-lineage cells from cervical cancer patients
- IMR90: fetal lung fibroblasts
- K562: mesoderm-lineage cells from individuals with leukemia
- NHEK: epidermal keratinocytes

TargetFinder identified active enhancers (3000 bp) and promoters (2000 bp) within each cell line by integrating ENCODE and Roadmap Epigenomics annotations. Additionally, using high-resolution genome-wide Hi-C data, each enhancer-promoter was annotated as either an interaction (positive samples) or non-interaction (negative samples). To ensure robust training, we adopted a meticulous sampling strategy: 20 negative samples were selected for every positive sample, maintaining a consistent 1:20 ratio of positive to negative samples across all cell lines. Both positive and negative samples were curated to reflect similar enhancer-promoter distance distributions. Detail information on the datasets for each cell line is provide in Table 2.

**Table 2.** Dataset information for each cell.

Cell line	Positive samples	Negative samples
GM12878	2113	42 200
HUVEC	1524	30 400
HeLa-S3	1740	34 800
IMR90	1254	25 000
K562	1977	39 500
NHEK	1291	25 600

The original imbalanced dataset is split into the training set (90%) and test set (10%). From the training set, 5% was further set aside for validation to assess model performance and prevent overfitting. To address the inherent class imbalance in our dataset, we employed two distinct strategies: data augmentation and class-weighted loss functions. These approaches were evaluated to identify the more effective method.

#### 2.1.1 Data augmentation

To address the imbalance between positive and negative samples, we employed a data augmentation technique inspired by Mao *et al.* (2017). Positive EPI samples were augmented by generating multiple slightly modified versions of each sample. Specifically, we shifted the genomic sequences of enhancers (3000 bp) and promoters (2000 bp) upstream or downstream by 10–50 base pairs. These shifts were carefully designed to preserve the biological relevance of the sequences while introducing variability.

By creating multiple variations of the positive class, this method not only increased the representation of positive samples but also enriched the dataset with biologically meaningful diversity. This enhanced variability was expected to improve the model’s ability to generalize and recognize subtle patterns in the interactions, addressing the limitations posed by the original 1:20 class imbalance.

#### 2.1.2 Class-weighted loss function

Another approach to handle the data imbalance, we implemented a class-weighted loss function. This technique assigned a higher weight to the underrepresented positive class during model training, ensuring that the model placed greater emphasis on accurately predicting positive interactions. Unlike data augmentation, which increases the number of positive samples, the class-weighted loss function directly modified the optimization process to reduce the influence of the dominant negative class.

### 2.2 Model structure

**Figure 1.**
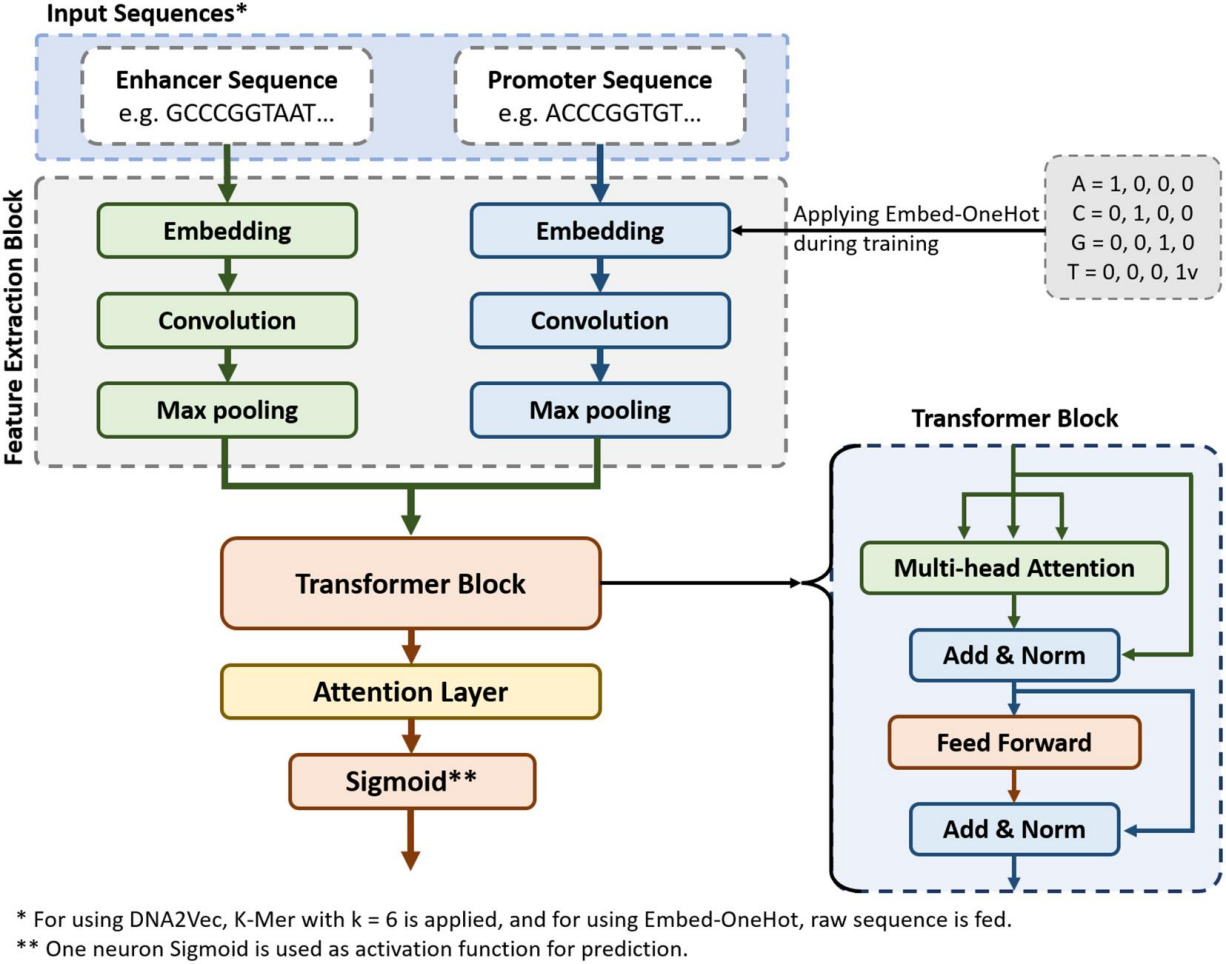
Schematic of the DeepEPI model for predicting enhancer-promoter interactions. The model input begins with separate enhancer and promoter sequences, each processed through embedding and convolutional layers to extract local features. These features are refined by max pooling, preparing the data for the transformer-block. The core of the model, the transformer-block, utilizes multihead attention to discern complex patterns and interactions. Add and Norm steps follow both the attention and feed-forward layers to maintain stability in the learning process. The attention layer strategically directs the model’s focus to critical segments, unveiling the interplay between regulatory elements. The final prediction is made by a neuron with sigmoid activation, integrating the information to predict EPIs.

According to the schematic model of DeepEPI, depicted in Fig. 1, DeepEPI is a comprehensive model leveraging deep learning techniques to elucidate the intricate mechanisms governing EPIs. Of course, prior to utilizing the model on the data, a preprocessing step is performed on the sequences to prepare them for input into the model. DeepEPI integrates a CNN module and a Transformer-based architecture, specifically designed to capture complex spatial dependencies and long-range interactions within genomic sequences. This innovative approach enables us to efficiently dissect enhancer and promoter regions, shedding light on the regulatory dynamics underlying gene expression. Additionally, we introduce an Attention Layer (Pappas and Popescu-Belis 2017), facilitating the identification of salient regions within the encode sequences. This mechanism enables the model to focus on pivotal segments, offering insights into the regulatory elements orchestrating EPIs. To ensure robustness and generalization, we integrate batch normalization and dropout layers, mitigating overfitting and enhancing model performance. The following sections are dedicated to explaining the main components of the DeepEPI architecture.

#### 2.2.1 Preprocessing input sequences

In this study, we utilize both OneHot encoding and DNA2Vec (Ng 2017) techniques to represent genomic sequences. OneHot encoding characterizes each nucleotide base as a binary vector, aiding model interpretation but facing challenges such as lack of inter-feature dependencies and high storage consumption. In contrast, DNA2Vec methods encapsulate intricate relationships and similarities within DNA sequences, offering storage efficiency due to encoding during training, especially beneficial for sequences with long lengths.

To optimize storage resources akin to DNA2Vec encoding within the OneHot encoding approach, we introduce a novel strategy. We employ embedding layers to encode sequences as OneHot representations during training. To achieve this, we construct a dictionary comprising the four nucleotides (A, C, G, T) and annotate undetermined bases as “N,” along with their corresponding OneHot encodings. By utilizing an embedding layer to capture raw sequence information and the OneHot dictionary for encoding, we set the trainable parameter for the embedding layer to false, creating what we term embed-OneHot encoding. This approach helps address the challenge of high storage consumption, though it still faces the issue of lack of inter-feature dependencies. However, this encoding offers advantages in terms of reducing running time, memory consumption, and simpler interpretation. We implement both encoding to compare the result, we use dimension 100 for DNA2Vec like PEP-WORD encoding in Hong *et al.* (2020).

#### 2.2.2 Features extracting block

The process starts by encoding enhancer and promoter sequences using an embedding layer, and then utilizes one-dimensional convolutional layers to extract local features crucial for regulatory function. These convolutional layers (Li *et al.* 2022) encode the promoter and enhancer sequences into separate feature maps. Max-pooling layers are utilized to distill essential information, facilitating the effective fusion of enhancer and promoter representations. The output from both sequences is merged to form a unified representation. The formulas for the features extracting are described in equation (1), (2), and (3).


(1) $$E_{o}=\mathrm{MaxPooling}(\mathrm{Convolution}(\mathrm{Embedding}(E)))$$

(2) $$P_{o}=\mathrm{MaxPooling}(\mathrm{Convolution}(\mathrm{Embedding}(P)))$$

(3) $$M=\mathrm{Merge}(P_{o},E_{o})$$

*E* refers to the enhancer, which has 3000 bp, and *P* refers to the promoter, which has 2000 bp when fed into the model. *M* represents the merged output of the features extracted from and $E_{o}\;\mathrm{and}\;P_{o}$, containing information from both sequences.

#### 2.2.3 Transformer block

The transformer block plays a crucial role in capturing dependencies and interactions within EPIs. Inspired by the transformer architecture, originally proposed for natural language processing tasks, it has been effectively modified for genomic sequence analysis. Within the transformer block, the key mechanism is the multihead attention layer, allowing the model to focus on different parts of the input sequences simultaneously, enabling it to extract intricate dependencies within the enhancer and promoter regions. By attending to relevant features across the entire sequence, the multihead attention mechanism identifies critical regulatory motifs and patterns essential for EPI. Moreover, the Transformer block incorporates feed-forward neural networks and layer normalization, facilitating the extraction of higher-level representations and ensuring stable gradient flow during training. The feed-forward layers capture non-linear relationships within the data, enabling the model to discern complex regulatory signals inherent in EPIs (Han *et al.* 2020). Equation (4) shows transformer block’s output where represents the contextualized encoding of combined promoter-enhancer information.


(4) H=TransformerBlock(M)

#### 2.2.4 Attention layer

The hierarchical attention layer is applied to the transformer block output H to determine which parts of the features are most important for the final prediction. The formula for the attention is described in Equation (5).


(5) $$\sum_{i}{\mathrm{Softmax}((\tan(W_{h}\;h_{i}+b_{h}))}^{T}u_{c})h_{i}$$

Each row $h_{i}$ in the Transformer output H represents contextualized information. $W_{h}\;$is a learned weight matrix, and $b_{h}$ is a bias. The tanh function computes a hidden representation that reflects the importance of region i, and${\;u}_{c}\;$is a trainable context vector that helps determine which regions are important, the softmax is applied across all i, normalizing the attention weights. The summation over i ensures that the final representation S is a weighted sum of all regions.

### 2.3 Training phase and fine tuning

Regarding hyperparameter tuning, certain parameters, such as the convolution filter and kernel size, pooling size, and stride, were initially chosen based on insights from previous research. For other parameters, an iterative approach was used. The model was first trained with a broad range of hyperparameters, selected based on empirical knowledge and literature, and then fine-tuned using a validation set. A grid search technique is employed to explore the parameter space and optimize performance. Table 3 reports the final hyperparameters result from this assessment.

**Table 3.** Hyperparameter of DeepEPI model.

Hyperparameter	Value
Convolution filter#	128
Convolution kernel size	40
Max pooling size and stride	20, 20
All dropout	0.5
Transformer heads#	16
Dense1 unit# in transformer-block	255
Dense2 unit# in transformer-block	128
Attention layer dim	246
Dense	1
Batch size	64

We implement DeepEPI using TensorFlow, leveraging a batch size of 64 samples for efficient processing. The cross-entropy loss function computes the error during training, facilitating model optimization. We employ the Adam optimization algorithm to update the neural network weights, enhancing convergence and performance. To mitigate overfitting, we incorporate batch normalization and dropout techniques with a random drop probability (*P* = .5) following the merge layer.

DeepEPI undergoes training in two distinct ways. Initially, it is trained on each cell line separately and subsequently evaluates the models individually. However, due to the presence of common features across multiple cells, we amalgamate the training datasets from all six cells into a unified dataset. Then, we train the DeepEPI on this consolidated dataset to capture shared features among the cell lines. This version is highlighted in plots in the result section with the suffix “best.” Following this, we conduct separate training sessions for each cell line, leveraging the insights gained from the collective training process.

### 2.4 Extraction of motifs and TFs interaction

In our study, we extract motifs and TFs interactions from the heads of the multihead attention layer in the transformer-block. Since the multihead attention layer is responsible for identifying feature dependencies, we focus our analysis on this layer. Once the model has been trained, we input positive test samples for each individual cell line into the model and capture the weights of the 16 heads to identify the most relevant regions between both sequences. Each head is 246 × 246, with 148 parts dedicated to enhancers and 98 parts allocated for promoters. So, our goal is to extract the motifs from the promoters and enhancers that correspond to the 246 parts of the heads. To achieve this, we apply and simulate CNN and max pooling techniques on the positive test sequences, allowing us to represent each part of the 246 heads in the PWM matrix and identify motif-like features. Convolutional kernels with a sliding window scan for relevant features, and max pooling helps capture the most important ones based on the trained weights. This process allows us to link the most representative motifs back to the attention heads. We then aggregate nucleotide occurrences to construct position weight matrices (PWMs), which represent nucleotide frequencies within each motif. To assess the promoter and enhancer dependency motifs, we utilize the weights of the heads to identify the 5 to 8 highest best scores across the average 16 heads. These scores form the basis for evaluating promoter and enhancer interactions. Finally, we identify TFs from the PWM matrices using the TOMTOM tool (Gupta *et al.* 2007) and HOCOMOCO Human v11 database with an *e*-value (<0.07) and *P* value (<.0001). Following this, we examine TFs interactions (physical, genetic, indirect) using the BioGRID tool (Oughtred *et al.* 2021) to validate the motifs interaction has identified by heads. You can find more detail in File 1, available as supplementary data at *Bioinformatics Advances* online. It should be noted that BioGRID reports are general, and TF-TF interactions are not cell line-specified.

### 2.5 Evaluation metrics

In our study, we encounter datasets characterized by extreme imbalance, wherein the distribution of positive and negative samples was significantly skewed. Consequently, we adopt evaluation metrics tailor to handle such imbalance classification scenarios: the area under the receiver operating characteristic curve (AUROC) (Hanley and McNeil 1982) and the area under the precision-recall curve (AUPR) (Davis and Goadrich 2006). The receiver operating characteristic curve, representing sensitivity against the false-positive rate, elucidates the trade-off between sensitivity and specificity across various classification thresholds. AUROC quantifies the overall discriminative power of the model, with a value closer to 1 indicating superior performance. Notably, AUROC remains robust irrespective of the imbalance in class distributions, rendering it a suitable measure for assessing models trained on imbalance datasets.

In contrast, the precision-recall curve delineates the balance between precision and recall, portraying the model’s ability to accurately identify positive instances while covering the entirety of positive cases. AUPR, the area under the precision-recall curve, signifies the model’s precision-recall trade-off, with a higher AUPR value indicative of better model performance. AUPR metric provides valuable insights, especially in scenarios where class imbalances heavily influence classification outcomes. By leveraging AUROC and AUPR metrics, we ensure thorough assessment of our model’s performance, taking into consideration the complexities of imbalance binary classification tasks.

## 3 Results

In this section, we present findings in five sections. First, we compare the effectiveness of the Class-Weighted loss function against data augmentation. Second, we evaluate the contribution of each model block to overall performance. Third, we assess the impact of three different encoding methods OneHot, Embed-OneHot, and DNA2Vec on model performance and resource consumption. Fourth, we compare DeepEPI with other existing tools. Finally, we provide a detailed analysis of transcription factor (TF) extraction and their interactions.

### 3.1 Evaluate class-weighted loss function *versus* data augmentation

To address the inherent class imbalance in our dataset, we evaluated two independent strategies: data augmentation and class-weighted loss functions. The results of the DeepEPI model trained on augmentation datasets for the HUVEC cell line demonstrated that the augmentation strategy effectively enhanced accuracy. Specifically, for a 10 bp shift, the model achieved an AUC of 0.9289 and an AUPR of 0.6695; for a 25 bp shift, the AUC was 0.9215 and the AUPR was 0.6790; and for a 50 bp shift, the model achieved the best results, with an AUC of 0.935 and an AUPR of 0.705. This indicates that the 50 bp augmentation introduced biologically meaningful variations, enhancing the model’s ability to generalize and recognize subtle patterns in EPIs.

DeepEPI model trained with class-weighted loss function approach achieved an AUC of 0.888 and an AUPR of 0.452 for the HUVEC cell line, significantly lower than the results obtained with the data augmentation strategy. While the class-weighted loss function effectively handling class imbalance, it failed to address the lack of variability in the training data, which limited its ability to improve accuracy.

The results demonstrate that data augmentation, particularly with the 50 bp shift, was superior in mitigating class imbalance while enriching the dataset with biologically relevant diversity. By increasing variability, this approach allowed the model to learn more robust and generalizable patterns, resulting in improved predictive accuracy compared to the class-weighted loss function. These findings highlight the importance of enhancing data diversity in training datasets for achieving better model generalization and handling class imbalance.

### 3.2 Evaluating model performance in three steps

To discern which model block had the greatest impact on accuracy, we evaluated the model in three stages. We began with a simple architecture comprising a convolutional layer, max pooling, and a fully connected layer as the baseline. To enhance performance, we then introduced a transformer block, known for its strength in handling sequential data by capturing long-range dependencies that traditional layers may overlook. This addition, followed by the fully connected layer, led to significant improvements in both ROC and PR metrics. Notably, PR accuracy saw a more substantial increase, highlighting the transformer’s ability to learn intricate patterns within sequences and improve feature extraction for more precise predictions.

**Figure 2.**
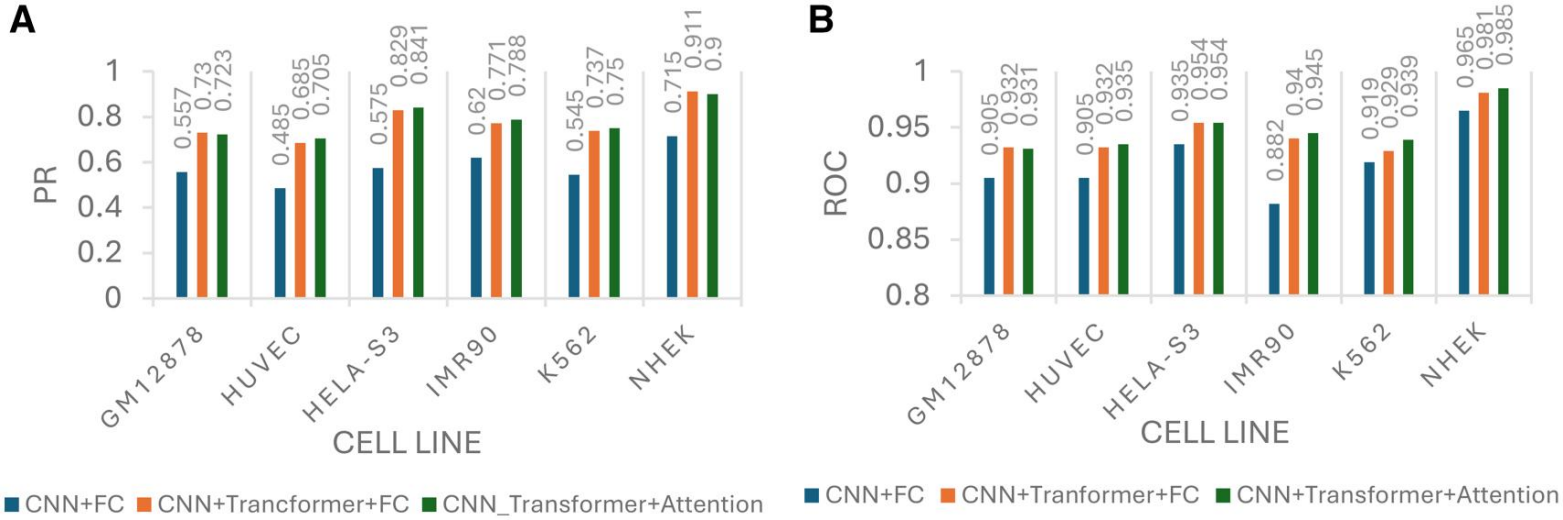
Bar chart depicting the comparison of AUPR and AUROC across six different cell lines with embed-OneHot coding in three steps to analysis effect of each block in the model. (A) Bar chart depicting AUPR for each cell line. (B) Bar chart illustrating AUROC for each cell line.

In the final step, we replaced the fully connected layer with an attention mechanism, which resulted in slightly higher accuracy across most cell types, except for GM12878 in both PR and ROC, and NHEK in PR. This transition demonstrated that attention layers are slightly more effective than fully connected layers in capturing relevant features for final decision-making. The chart in Fig. 2 visualizes the accuracy progression through all three stages of the model, with each step utilizing embed-OneHot encoding.

### 3.3 Encoding methods comparison

We present a comprehensive comparative analysis of three encoding methodologies: DNA2Vec, OneHot and Embed-OneHot encoding. Our study evaluates these methods across six distinct cell lines, while each encoding method is trained independently for each cell line.

**Figure 3.**
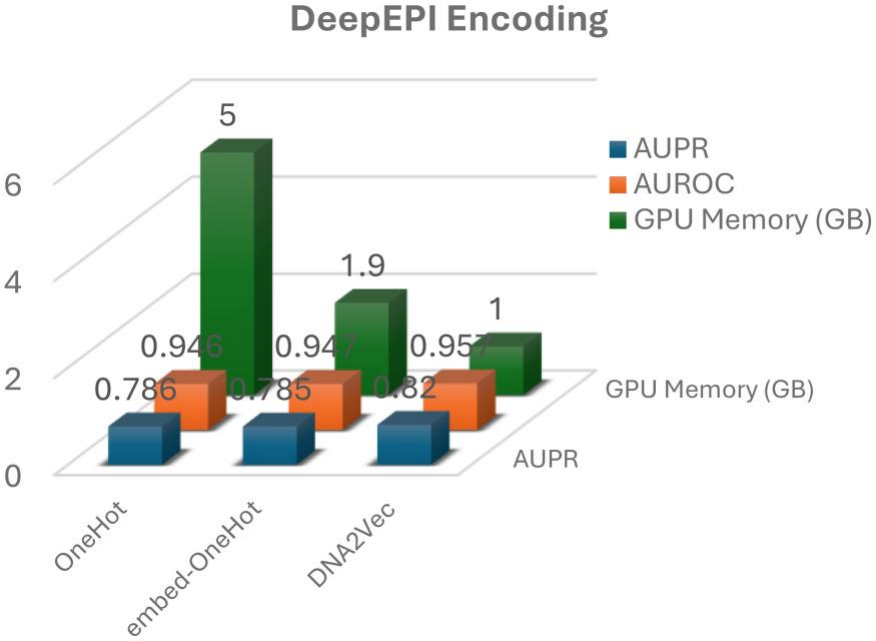
Bar chart comparing AUPR and AUROC across six different cell lines using three encoding methods. The results show that, on average, DNA2Vec achieves the highest AUPR and AUROC across all cell lines. While Embed-OneHot encoding reduces GPU memory consumption compared to OneHot encoding, it still requires more memory than DNA2Vec. However, Embed-OneHot enhances model interpretability, presenting a trade-off between interpretability and overall predictive performance.

Figure 3 illustrates the performance of the three encoding approaches traditional OneHot, Embed-OneHot, and DNA2Vec under identical model conditions (DeepEPI with a batch size of 32) using a GeForce NVIDIA GPU with 8 GB of memory. The results indicate that DNA2Vec achieves the highest accuracy (AUROC and AUPR); however, its learning-based nature increases training time and reduces interpretability.

In contrast, Embed-OneHot encoding strikes a balance between interpretability and performance by eliminating the need for additional learning during training. This method dynamically generates OneHot encodings on-the-fly (Abadi *et al.* 2016) significantly reducing GPU memory consumption. As shown in Fig. 3, Embed-OneHot encoding required ∼1.9 GB of GPU memory, representing a reduction of nearly 3 GB compared to traditional OneHot encoding. Additionally, it resolved GPU memory errors encountered with larger batch sizes. Larger batch sizes not only circumvent memory limitations but also accelerate model convergence, thereby reducing overall training time. Details, such as GPU/CPU consumption monitoring during the training phase using the Wandb tool (Biewald, 2020), can be found in File 2, available as supplementary data at *Bioinformatics Advances* online.

These findings underscore the utility of Embed-OneHot encoding in scenarios that prioritize interpretability and resource efficiency. Conversely, DNA2Vec is more suitable for tasks that demand maximal accuracy, despite its increased computational overhead and reduced interpretability.

### 3.4 Tools comparison

**Figure 4.**
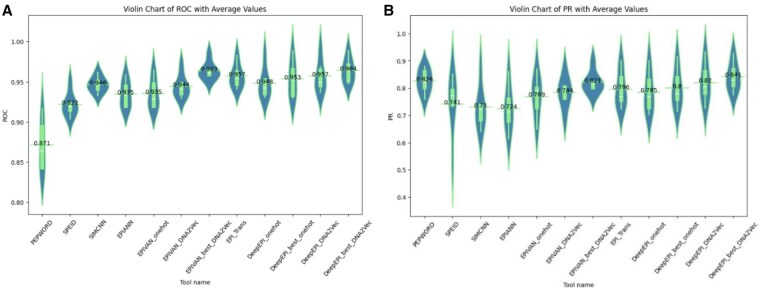
Violin chart depicting AUROC and AUPR, comparing DeepEPI with state-of-the-art tools across six cell lines. (A) Violin chart of AUROC with average values. (B) Violin chart of AUPR with average values. In Table 1, we provide references relevant to each tool. However, since we used the same dataset and augmentation method as in the EPIANN study, we directly report the accuracy of PEPWORD from it.

In this section, we compare the accuracy of previous tools with DeepEPI using different training approaches and encoding states. Figure 4 demonstrates violin charts comparing tools using both AUPR and AUROC accuracy metrics. As previously explained at the section “Training phase,” DeepEPI model with embed-OneHot encoding (label DeepEPI_best_onehot in plots) and DeepEPI model with DNA2Vec encoding (label DeepEPI_best_DNA2Vec in plots) are trained on the entire dataset comprising six cell types and subsequently trained on each dataset separately. As depicted in the chart, DeepEPI achieves higher accuracy with the DeepEPI_best models compared to other DeepEPI models trained solely on individual cell types in two types of encoding. This superiority can be attributed to the training on the entire dataset, enabling the models to capture common features. Additionally, as illustrated in Fig. 4A, DeepEPI_best_DNA2Vec has achieved a superior average AUROC compared to previous tools and closely aligning with the AUROC of the EPIVAN_best_DNA2Vec tool and showing 0.7% improvement compared to the EPI-Trans tool. Moreover, the DeepEPI_best_onehot tool exhibits a higher average AUROC compared to all preceding models except EPIVAN_best_DNA2Vec, with an AUROC drop of ∼1.03%. Figure 4B demonstrates that DeepEPI_best_DNA2Vec achieves superior average AUPR compared to previous tools and showing 5.65% improvement over the recent EPI-Trans tool. It also closely aligns with the AUPR of the PEPWORD tool, with an improvement of ∼1.82%. Moreover, the DeepEPI_best_onehot tool demonstrates a higher average AUPR compared to all preceding models except EPIVAN_best_DNA2Vec and PEPWORD, with an AUPR drop of ∼2.6% in EPIVAN_best_DNA2Vec and about 3.1% in PEPWORD.

However, DeepEPI_best_onehot offers the benefit of making model interpretation easier because of its simpler encoding and showing a 4% increase in AUPR and a 1.9% improvement in AUROC compared to the previous EPIVAN-onehot tool. Additionally, compared to EPIANN, which extracted TF interactions using an attention mechanism, DeepEPI demonstrates an average increase of 1.9% in AUROC and 10.2% in AUPR.

As our study’s main goal is model interpretation, we will concentrate on OneHot encoding in the interpretability section. If you prioritize accuracy, DeepEPI_best_DNA2Vec is the superior choice. For more details about the accuracy of each tool in separate cell lines, please refer to File 3, available as supplementary data at *Bioinformatics Advances* online.

### 3.5 TF identification and TF interactions

In this section, we first extract TFs and then identify their interactions. One of the greatest advantages of using the transformer layer is its multihead attention mechanism, which enables the extraction of both TFs and TF interactions. We extracted 158 TFs in promoters and 154 TFs in enhancers through 16 heads of the multihead attention layer, as explained in motifs and TFs extraction interaction section. File 4, available as supplementary data at *Bioinformatics Advances* online, depicts eight heatmap figures of extracted TFs from promoters and enhancers, which consist of the most important attention scores in average heads. Based on these heatmaps, the TFs commonly found across six cells in promoters include: EGR1, EGR2, KLF15, KLF6, KLF3, SP1, SP2, SP3, SP4, PATZ1, MAZ, RXRA, THAP1, Vezf1, WT1, ZBT17, ZN263, ZN341, ZN467, and ZN563. Similarly, The TFs commonly found in heatmaps across six cells in enhancers include: HXC10, IRX2, MNX1, NR1D1, RXRA, ZN394, and ZN563. You can view the logos of some TFs and the corresponding TSV and graphical HTML files containing the extracted TFs in File 5, available as supplementary data at *Bioinformatics Advances* online.

Additionally, motif frequency can be an important indicator of motif relevance, especially for widely occurring motifs like SP1, which are known to appear multiple times in promoter regions. We performed a new analysis of motif frequencies in both promoter and enhancer regions across six human cell lines. Our results show that SP1 is among the most frequently observed transcription factors in promoter regions, particularly in the following cell lines (in descending order): GM12878, HeLa, HUVEC, IMR90, K562, and NHEK. These findings are consistent with prior studies such as (Dynan and Tjian 1983), which reported SP1 binding upstream of the SV40 early promoter, helping validate the biological relevance of our observations.

Other frequently detected transcription factors in promoter regions include RXRA (in GM12878, HUVEC, IMR90, K562, and NHEK), SP2, SP3, KLF6, and ZNF341 (across all six cell lines), as well as NR1D1 (in GM12878, HeLa, HUVEC, IMR90, and K562). The high frequency of SP/KLF family members (e.g. SP2, SP3, and KLF6) supports their known role as GC-box-binding TFs regulating a broad array of genes, as discussed by Suske *et al.* (2005).

In enhancer regions, our analysis shows that motifs for MNX1 (in GM12878, HeLa, HUVEC, K562, and NHEK), TLX1 and RXRA (in GM12878, HUVEC, IMR90, K562, and NHEK), and GATA1 (in K562 and HeLa) are among the most prevalent. The enrichment of GATA1 in K562, an erythroid lineage cell line, is in line with findings from Romano et al. (2020), who demonstrated GATA1 binding at a stage-specific super-enhancer in human erythroid progenitors. A detailed heatmap illustrating these motif frequencies across all cell lines and genomic regions is included in File 6, available as supplementary data at *Bioinformatics Advances* online.

**Figure 5.**
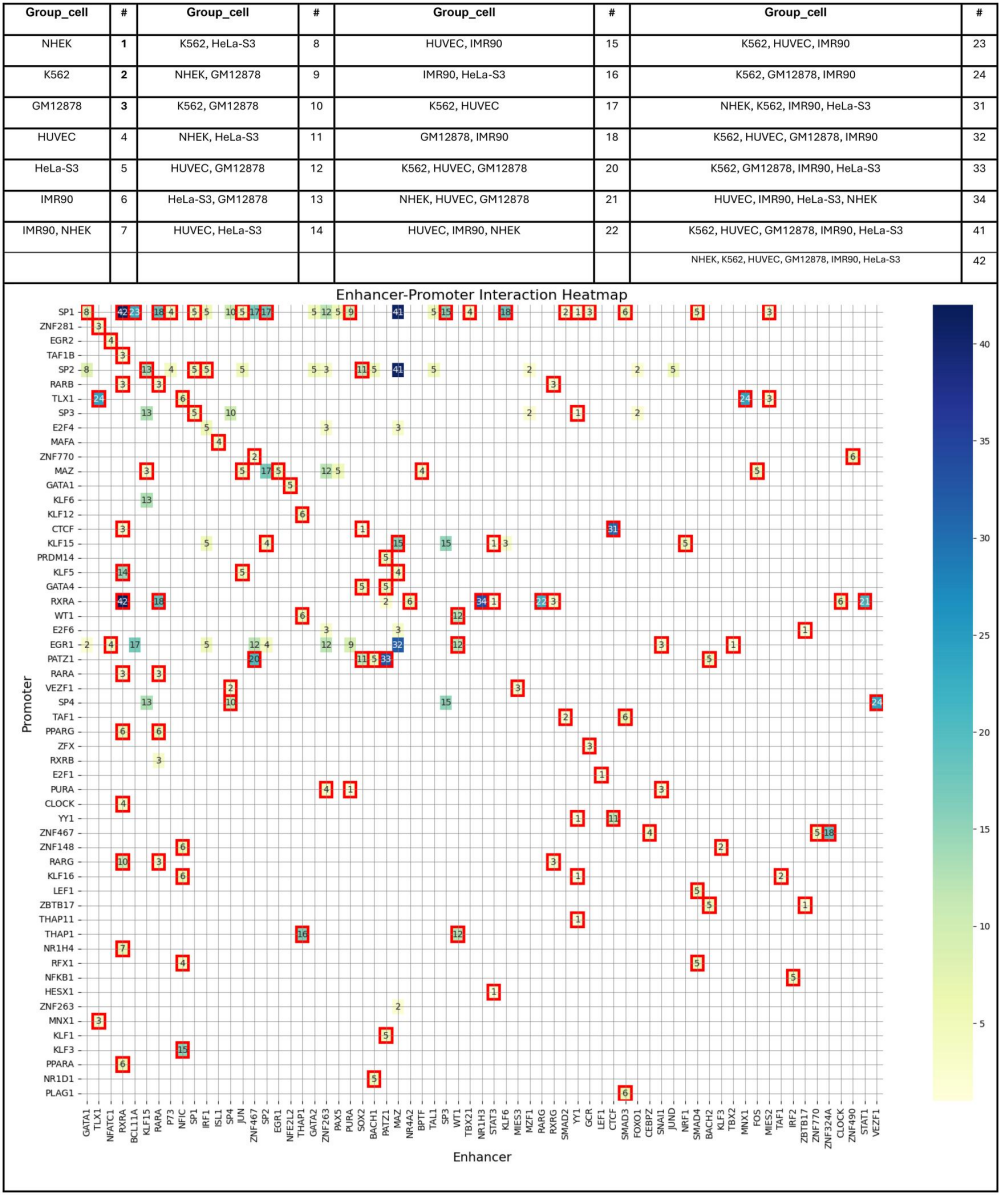
Illustrates transcription factor (TF) interactions across different cell types in enhancer and promoter. Above the figure, there is a table labeled Group_Cell, which lists the cell types corresponding to specific numbers. These numbers indicate the group of cell types where each TF interaction is detected in the heatmap. For example, the entry “NHEK, K562, HUVEC, GM12878, IMR90, HeLa-S” under Group_Cell with a corresponding “#” value of 42 indicates that the interactions in the figure associated with this number occur in these listed cell lines. Deeper shades of blue indicating more prevalent interactions. The numbers on the heatmap denote the count of cells showing specific TF interactions, with higher values indicating a more universal presence across different cell types. A red outline around an interaction indicates a physical interaction (PI) identified by BioGRID.

We identified 172 TF interactions using a 16-head multihead attention layer across six cells and 75.6% of these interactions are supported as Physical Interactions (PI) in BioGRID. Figure 5 demonstrates the map of TF interactions between promoters and enhancers. The numbers in the map correspond to a table and represent specific categories of cells, indicating where TF interactions were found. Each number denotes the magnitude of interactions contributing to multiple cells; higher numbers indicate greater involvement across cells. For example, we identified RXRA–RXRA and RXRA–SP1 interactions with a number of 42, indicating that these interactions were observed in all six cells. In this notation, the left TF represents the enhancer, while the right one represents the promoter. We found that RXRA in the enhancer contributed to 12 interactions, indicating a higher involvement compared to other TFs in the enhancer. Similarly, SP1 in the promoter contributed to 26 interactions, demonstrating its greater contribution compared to other TFs. Also, we create tables with the key interactions validated for each cell type and the type of interactions (physical, genetic, indirect) as identified in BioGRID. For instance, in the NHEK cell line, interactions such as SOX2—CTCF were validated as physical interactions. These results will highlight the biological significance of the identified TF-TF interactions and enhance the depth of our analysis. You can see details in File 7, available as supplementary data at *Bioinformatics Advances* online. This supplementary includes six tables that detail the interactions across six cell lines, with the number of motifs and the type of each interaction clearly described.

Given the critical role of CTCF-CTCF (Ren *et al.* 2017) interaction in chromatin organization and gene regulation, we conducted a thorough investigation across all regions of the attention heads, not limiting our analysis to the top 5 to 8 most important scores like TF identification and interactions section. This allowed us to comprehensively assess the presence of CTCF-CTCF interactions across different cell lines with its frequency. We successfully identified CTCF-CTCF interactions in four cell lines (NHEK, K562, IMR90, and HeLa) especially with higher frequency in IMR90 and K562 with weights close to the best weight heads. However, in GM12878 and HUVEC, CTCF motifs were only found in promoters, suggesting that CTCF-CTCF interactions may be weaker or absent in these cell lines’ enhancers. The detailed results, including motifs number, attention head weights, and e-values, are provided in the File 8, available as supplementary data at *Bioinformatics Advances* online for further reference.

### 3.6 Validation of TF binding sites in cell lines

To validate the identified transcription factors (TFs), we conducted a detailed analysis using the ENCODE database for the corresponding cell lines. The goal of this validation was to determine how many of the predicted TFs are biologically meaningful and likely play a role in gene expression. Our validation process involved:

- **Data Extraction**: For each relevant cell line, we retrieved publicly available DNase-seq and Chip-seq data from the ENCODE database, both filtered by the “TF target” category. DNase-seq data included computationally annotated footprints, which suggest potential TF binding sites based on protection patterns in accessible chromatin. Chip-seq data directly indicates TF–DNA binding events, based on immunoprecipitation of DNA fragments bound to specific TFs. We utilized both assays due to their complementary advantages and the varying availability of experimental data across TFs and cell lines.
  DNase-seq identifies regions of open chromatin, where TF binding is more likely to occur. Bound TFs can leave “footprints” by protecting DNA from DNase cleavage, increasing the likelihood of in vivo TF binding at those sites. However, DNase footprints are not definitive, as similar patterns may result from other chromatin-associated factors or enzymatic bias. In contrast, Chip-seq offers more specific evidence but relies on high-quality antibodies and is not available for all TFs. By integrating DNase-seq footprints and Chip-seq peaks both targeting TFs, we performed a more reliable and comprehensive validation of the binding sites predicted by our motif analysis.
  Additionally, due to limited ENCODE coverage, validation was performed in four cell lines with sufficient data HeLa-S3, IMR90, GM12878, and K562. Validation was not possible for HUVEC and NHEK due to insufficient data in Encode for these cell lines.
- **Overlap Analysis:** We used Bedtools to quantify the overlap between our enhancers and promoters regions and the genomic coordinates of ENCODE experimental peaks, derived from both DNase-seq footprints and Chip-seq data.

For example, in the HeLa-S3 cell line, among all the transcription factors (TFs) we identified, ENCODE data was available to validate 84% of them. Within this subset, 98.4% showed significant overlap with our enhancer regions, providing strong support for the biological relevance of our TFs predictions.

The remaining 16% of TFs were not available in ENCODE. Within this subset, 58.3% lack any TF binding data in ENCODE, reflecting a gap in empirical evidence rather than a confirmed absence of binding in HeLa-S3; these TFs may be characterized in future studies. The remaining 41.7% have been assayed in HeLa-S3 but showed no evidence of binding, suggesting they may not play a functional role in this specific cell line. Overall, our findings indicate that most TFs identified by our model correspond to biologically meaningful binding events, underscoring the functional significance and interpretability of the sequence features captured.

**Figure 6.**
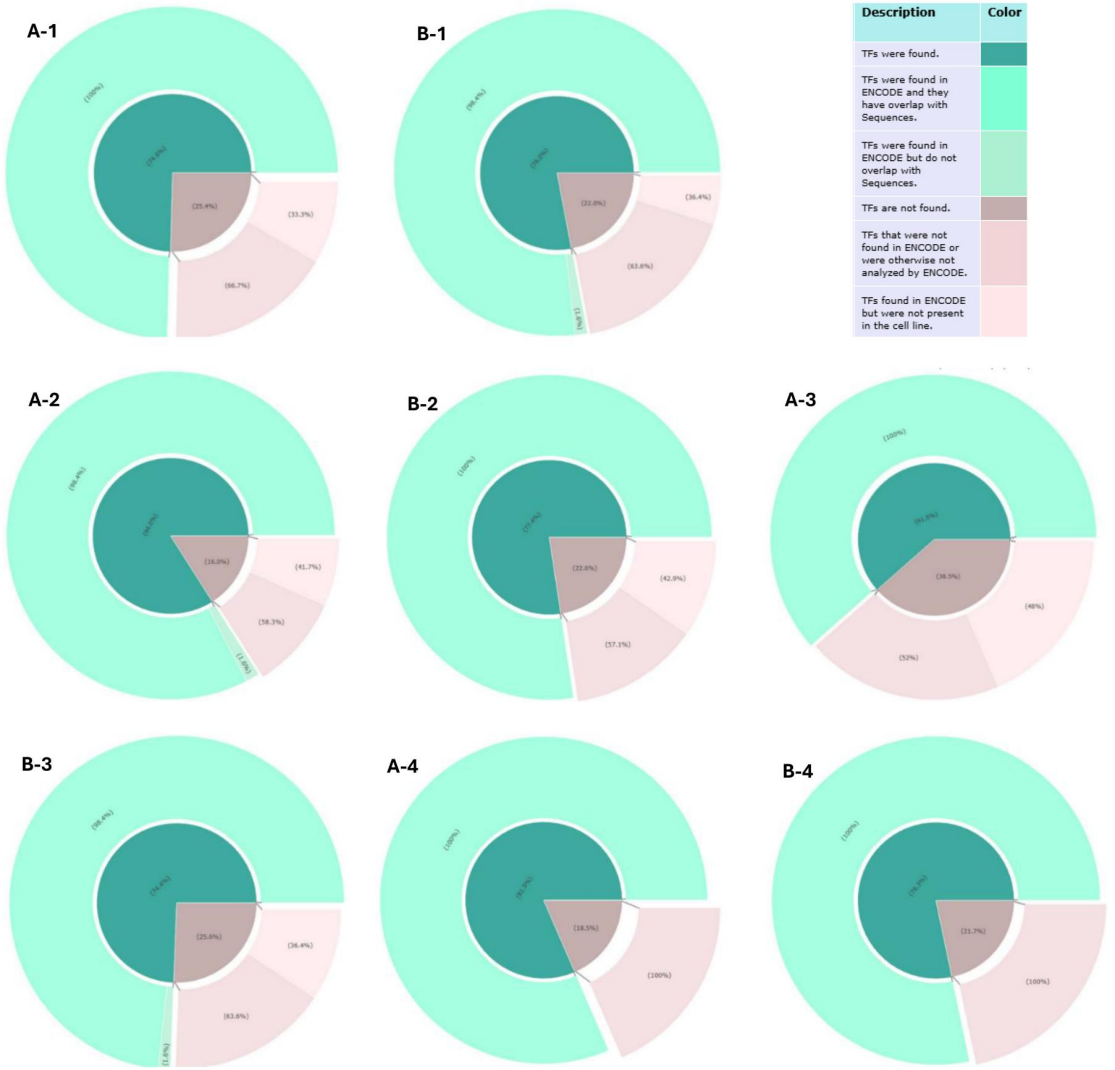
Validation results of transcription factor (TF) analysis in enhancer and promoter regions across four human cell lines HeLa-S3, IMR90, GM12878, and K562, using the ENCODE database, based on Chip-seq and DNase-seq assays. A-1: TF analysis results for the enhancers in the GM12878 cell line: Among all TFs identified by our model, 74.6% were available in ENCODE for validation, and all of them showed overlap with our sequences. Among the remaining 25.4%, 66.7% were not available by ENCODE in any cell line, while the other 33.3% had been analyzed but showed no binding evidence in GM12878, possibly indicating that these TFs do not play a regulatory role in this cell line. B-1: TF analysis results for the Promoters in GM12878 cell line. Among all TFs identified by our model, 78% were available in ENCODE for validation and 98.4 have overlap with our sequences. Among the remaining 22%, 63.6% were not available by ENCODE in any cell line, while the other 36.4% had been analyzed but showed no binding evidence in GM12878, possibly indicating that these TFs do not play a regulatory role in this cell line. A-2: TF analysis results for the Enhancers in HeLa-S3 cell line. Among all TFs identified by our model, 84% were available in ENCODE for validation and 98.4 have overlap with our sequences. Among the remaining 16%, 58.3% were not available by ENCODE in any cell line, while the other 41.7% had been analyzed but showed no binding evidence in HeLa-S3, possibly indicating that these TFs do not play a regulatory role in this cell line. B-2: TF analysis results for the Promoters in HeLa-S3 cell line. Among all TFs identified by our model, 77.4% were available in ENCODE for validation, and all of them showed overlap with our sequences. Among the remaining 22.6%, 57.1% were not available by ENCODE in any cell line, while the other 42.9% had been analyzed but showed no binding evidence in HeLa-S3, possibly indicating that these TFs do not play a regulatory role in this cell line. A-3: TF analysis results for the Enhancers in IMR90 cell line. Among all TFs identified by our model, 61.5% were available in ENCODE for validation, and all of them showed overlap with our sequences. Among the remaining 38.5%, 52% were not available by ENCODE in any cell line, while the other 48% had been analyzed but showed no binding evidence in IMR90, possibly indicating that these TFs do not play a regulatory role in this cell line. B-3: TF analysis results for the Promoters in IMR90 cell line. Among all TFs identified by our model, 74.4% were available in ENCODE for validation and 98.4 have overlap with our sequences. Among the remaining 2598.4e not available by ENCODE in any cell line, while the other 36.4% had been analyzed but showed no binding evidence in IMR90, possibly indicating that these TFs do not play a regulatory role in this cell line. A-4: TF analysis results for the Enhancers in K562 cell line. Among all TFs identified by our model, 81.5% were available in ENCODE for validation, and all of them showed overlap with our sequences. Among the remaining 18.5%, 100% were not available by ENCODE in any cell line, suggesting a lack of available data rather than absence of binding. B-4: TF analysis results for the Promoters in K562 cell line. Among all TFs identified by our model, 78.3% were available in ENCODE for validation, and all of them showed overlap with our sequences. Among the remaining 21.7%, 100% were not available by ENCODE in any cell line, suggesting a lack of available data rather than absence of binding.

Additional information from our analysis includes overlap-related metrics, such as the average distance from the center of each TF binding site to the center of its associated sequence, as well as the relative position of the TF site within the sequence. Statistical measures including the mean, standard deviation, maximum, and first quartile are reported to describe the distribution of TF binding sites across sequence regions. These positions are normalized from 0 (start of the sequence) to 1 (end of the sequence), indicating where within the sequence the TFs tend to bind. Additionally, an Excel file summarizing TF validation results, indicating which TFs are supported by Chip-seq and which by DNase-seq is provided in File 9, available as supplementary data at *Bioinformatics Advances* online. Figure 6 displays the validation results TFs analysis in enhancer and promoter across HeLa-S3, IMR90, GM12878, and K562 cell lines.

Furthermore, an analysis of transcription factor (TF) binding sites within enhancers revealed distinct enrichment patterns across different cell lines. For the GM12878 cell line, the most abundant TF binding sites, in descending order, were observed for FOXD3, POU3F3, BRCA1, AIRE, SPIC, and RXRA. In HeLa-S3 cells, the dominant TF binding sites belonged to PATZ1, THRA, BACH1, SP1, CTCF, ZNF350, AIRE, and NFATC1. For the IMR90 cell line, CTCF, MAZ, ZNF263, KLF9, ZFX, and SP3 exhibited the highest number of binding sites. Lastly, in K562 cells, the most frequently observed TF binding sites were associated with TAF1, CTCF, PATZ1, SP4, MAZ, SP2, and MXI1.

Similarly, within promoter regions, specific TFs demonstrated prominent binding activity. In GM12878 cells, the most common TF binding sites were for ZBTB17, SP1, CTCF, BCL11A, NANOG, and KLF16. For HeLa-S3 cells, THRA, SP1, SP4, SP2, KLF6, KLF5, and BRCA1 showed the highest number of binding sites. In IMR90 cells, SP4, CTCF, SP2, ZNF148, KLF9, MAZ, and SP1 were the most frequently observed. A comprehensive list of additional TFs with the most binding sites in both enhancers and promoters can be found in File 9, available as supplementary data at *Bioinformatics Advances* online within the folder named Heatmaps.

### 3.7 Biological significance of recovered TF interactions

As mentioned in the previous sections, we aim to narrow the search scope for TF interactions to guide future experimental work in this work. Due to the current limitations in experimental resources for validating TF interactions within enhancer-promoter regions, direct comparison of our TF-TF interaction results remains challenging. However, the interactions identified in our study are biologically meaningful and supported by prior research, particularly in the context of EPIs. This section highlights some interactions that present novel hypotheses regarding their potential role in EPI.

- **SOX2–CTCF**: The BioGRID database and (Göös *et al.* 2022) provide experimental evidence of a physical interaction between SOX2 and CTCF in humans. This raises the hypothesis of a potential role in EPI via CTCF-mediated chromatin loops. Chakraborty *et al.* (Chakraborty et al., 2023) highlight the role of CTCF-mediated chromatin loops in EPIs while also noting that some interactions occur independently of CTCF. Further studies are needed to clarify impact of SOX2-CTCF interactions in enhancer promoter.
- **RXRA–PPAR and RXRA–PPARG**: RXRA forms heterodimers with PPARA and PPARG, which regulate lipid metabolism, energy homeostasis, and adipogenesis. These interactions highlight RXRA’s central role in gene regulation at enhancer and promoter regions (Halstead *et al.* 2017).
- **RAR–RXR Heterodimers (RARG–RXRA, RARA–RXRA, RARB–RXRA)**: RAR–RXR complexes play a crucial role in regulating gene transcription by binding to specific promoter and enhancer regions in the DNA in response to retinoic acid. This interaction at regulatory elements is essential for modulating the expression of genes involved in developmental processes, highlighting the significance of RAR–RXR-mediated control at promoter and enhancer sites to ensure proper developmental outcomes (Le Maire *et al.* 2020).
- **CTCF–CTCF:** CTCF-CTCF interactions facilitate chromatin looping, bringing enhancers and promoters together to regulate gene expression (Ren *et al.* 2017).
- **CTCF–YY1:** Based on BioGRID and (Donohoe *et al.* 2007), which show CTCF-YY1 interaction, and (Weintraub *et al.* 2017), which establish YY1 as a structural regulator of EPIs, we hypothesize that CTCF and YY1 may collaborate in chromatin organization, with potential implications for EPIs. CTCF anchors chromatin loops and defines insulated domains, while YY1 facilitates vs via dimerization. Their interaction at the Tsix locus suggests that YY1 may refine local interactions within CTCF-anchored loops, forming specialized hubs for transcriptional regulation.

## 4 Conclusion

In this article, we introduce a novel model named DeepEPI, designed to predict EPIs using only enhancer and promoter sequences. By incorporating a transformer block into DeepEPI, we aim to better capture hidden information features inherent in the sequences. Our experimental results, showcasing accuracy across six human cell lines, demonstrate that DeepEPI outperforms existing models utilizing DNA2Vec and embed-OneHot encoding. However, when employing embed-OneHot encoding, our method experiences a slight decrease in accuracy. Despite this, our emphasis on interpretability remains paramount in this study. Furthermore, DeepEPI offers the advantage of requiring less data storage space by leveraging an embedding layer for OneHot encoding. Notably, our approach can effectively extract TFs and TF interactions through the heads of the multihead attention layer within the transformer block, underscoring our model’s ability to focus on relevant TFs. In contrast to methods that only allow for creating certain models for sequence classification, our approach facilitates the discovery of meaningful TF interactions.

Our experimental findings underscore the substantial contribution of the transformer block to the model’s performance, with multihead attention playing a pivotal role in extracting TF interactions. This reinforces the capability of our model to capture both cell line-specific TFs and TFs common across cell lines. compared to the previous EPIVAN (Hong *et al.* 2020) tool, DeepEPI exhibits an average increase of 2.4% in AUPR and maintains the same AUROC, when using DNA2Vec encoding in both tools. DeepEPI also shows an average increase of 4% in AUPR and 1.9% in AUROC with OneHot encoding. DeepEPI is compared to the previous learning model, EPIANN, which extracts TF interactions using an attention mechanism, and as a result, DeepEPI shows an average increase of 1.9% in AUROC and 10.2% in AUPR. Additionally, DeepEPI narrows the search domain for finding TF interactions for future experimental approach.

The potential of the transformer block in biological applications has already been demonstrated. Looking ahead, future investigations will explore alternative encoding schemes, such as simple yet effective embed-OneHot encoding, *versus* more sophisticated approaches like word-embedding and DNA2Vec. For instance, adopting 2LK encoding (Akbari Rokn Abadi *et al.* 2023) could prove advantageous in subsequent studies. Therefore, the pursuit of an interpretable encoding method with high accuracy remains a promising avenue for future research endeavors. In the future as well, it is crucial to prioritize the analysis of the extracted TF interactions with the help of computational tools. This could significantly impact understanding and treatment of disease.

## Supplementary Material

vbaf221_Supplementary_Data

## Data Availability

The source code of DeepEPI is available at: https://github.com/nazanintbtb/DeepEPI.git.
