# Supplementary figures and images for: DeepEPI: CNN-transformer-based model for extracting TF interactions through predicting enhancer-promoter interactions
Source: Bioinform Adv. 2025 Sep 17;5(1):vbaf221. doi: 10.1093/bioadv/vbaf221 (PMC12478696; doi:10.1093/bioadv/vbaf221)

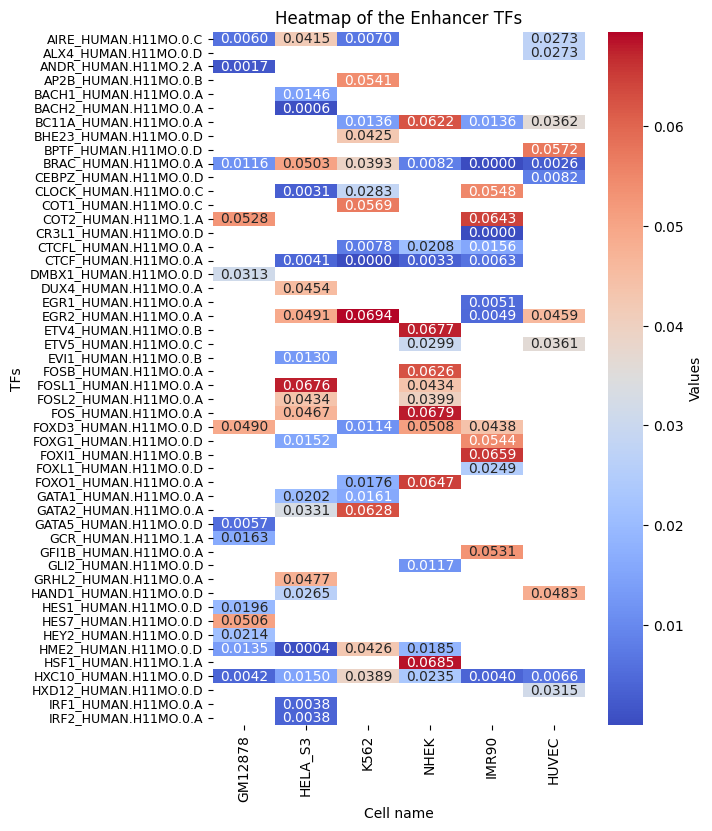

Supplement: vbaf221_Supplementary_Data [file vbaf221_supplementary_data.zip › Supplementary_file4/1-en.png]

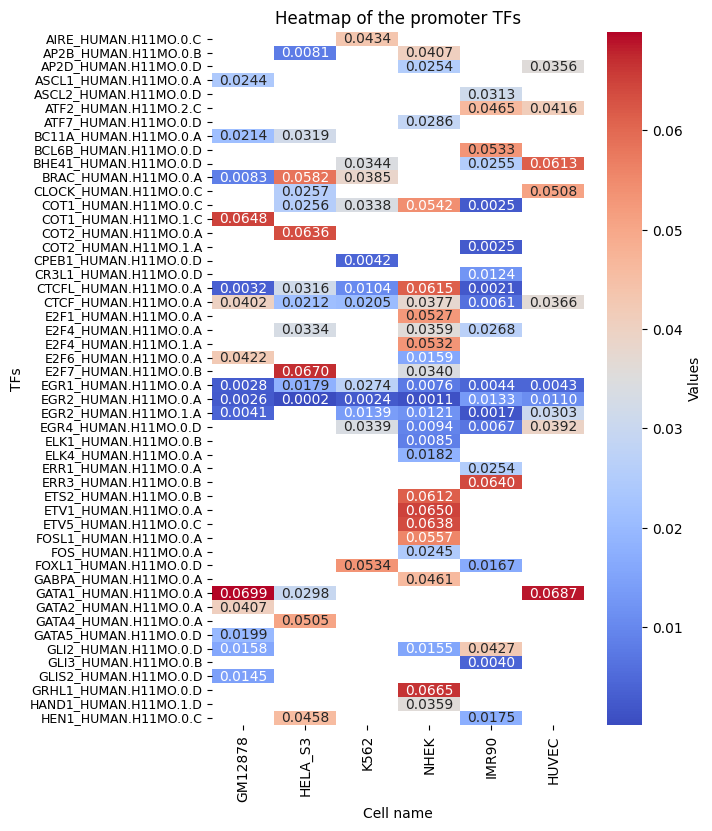

Supplement: vbaf221_Supplementary_Data [file vbaf221_supplementary_data.zip › Supplementary_file4/1-pro.png]

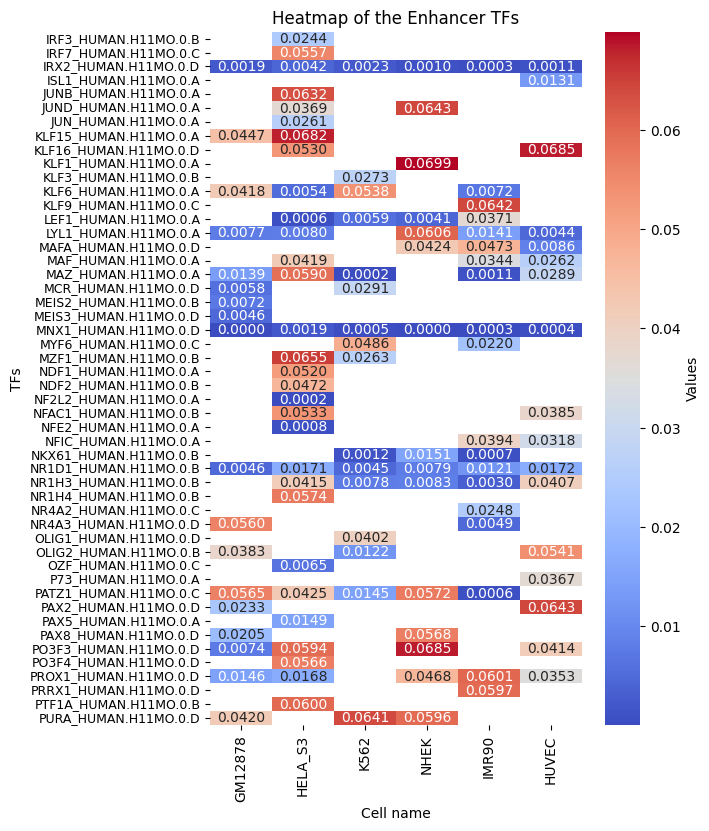

Supplement: vbaf221_Supplementary_Data [file vbaf221_supplementary_data.zip › Supplementary_file4/2-en.png]

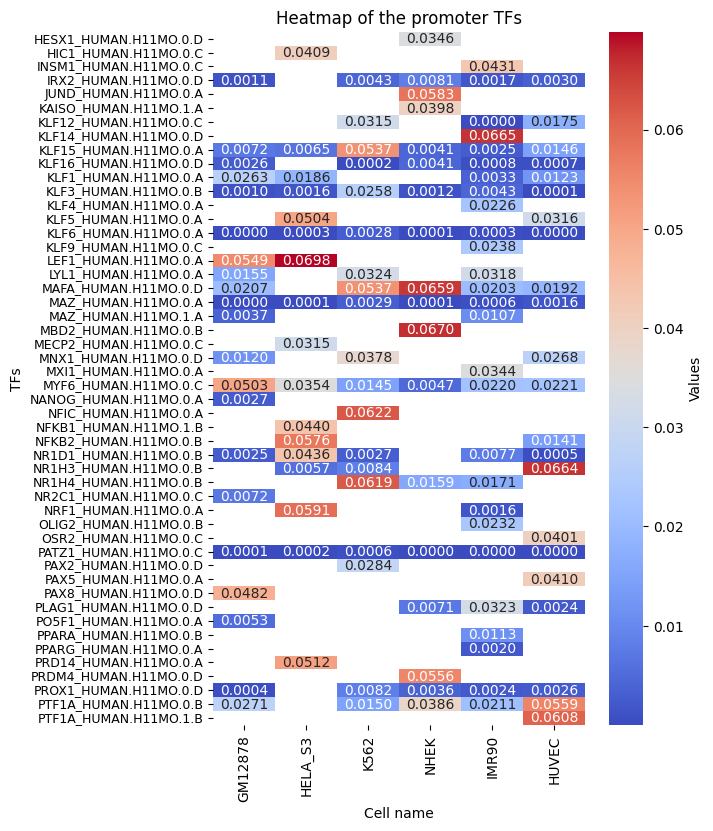

Supplement: vbaf221_Supplementary_Data [file vbaf221_supplementary_data.zip › Supplementary_file4/2-pro.png]

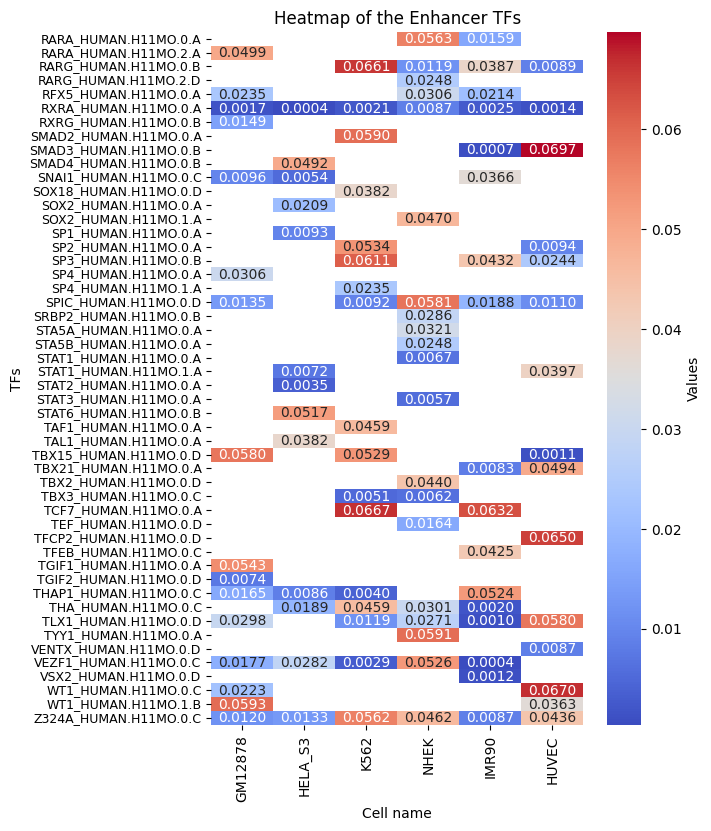

Supplement: vbaf221_Supplementary_Data [file vbaf221_supplementary_data.zip › Supplementary_file4/3-en.png]

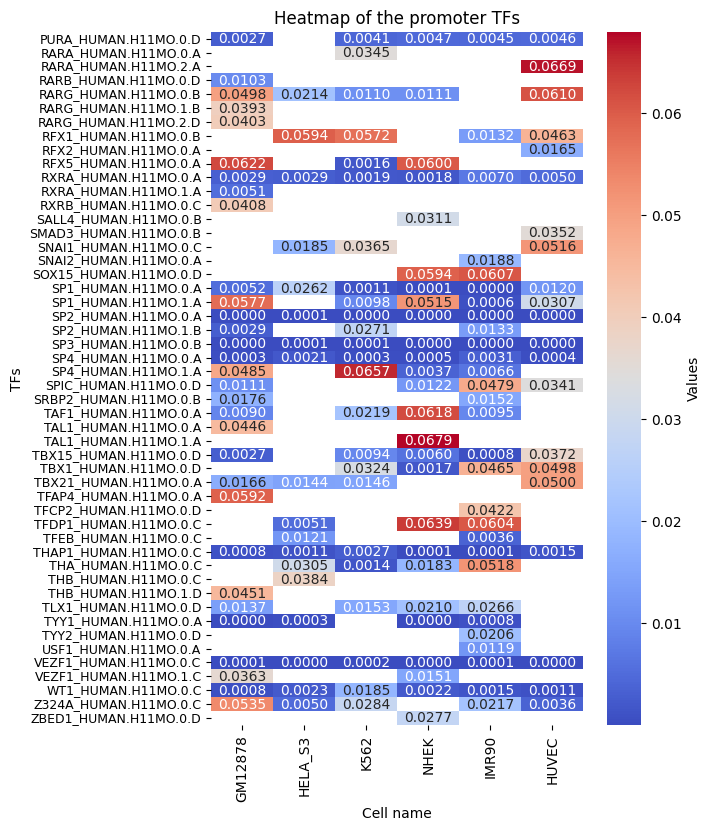

Supplement: vbaf221_Supplementary_Data [file vbaf221_supplementary_data.zip › Supplementary_file4/3-pro.png]

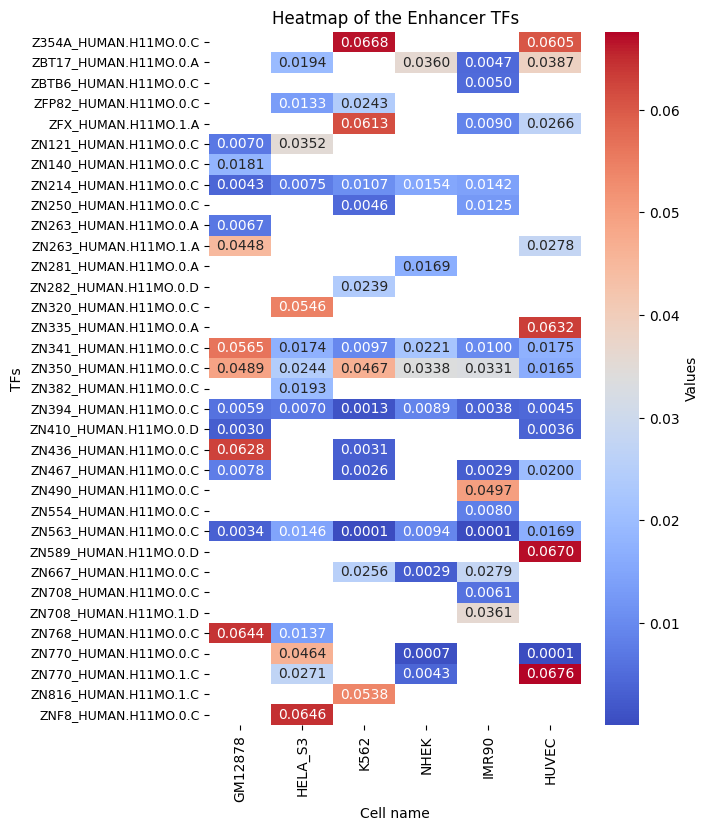

Supplement: vbaf221_Supplementary_Data [file vbaf221_supplementary_data.zip › Supplementary_file4/4-en.png]

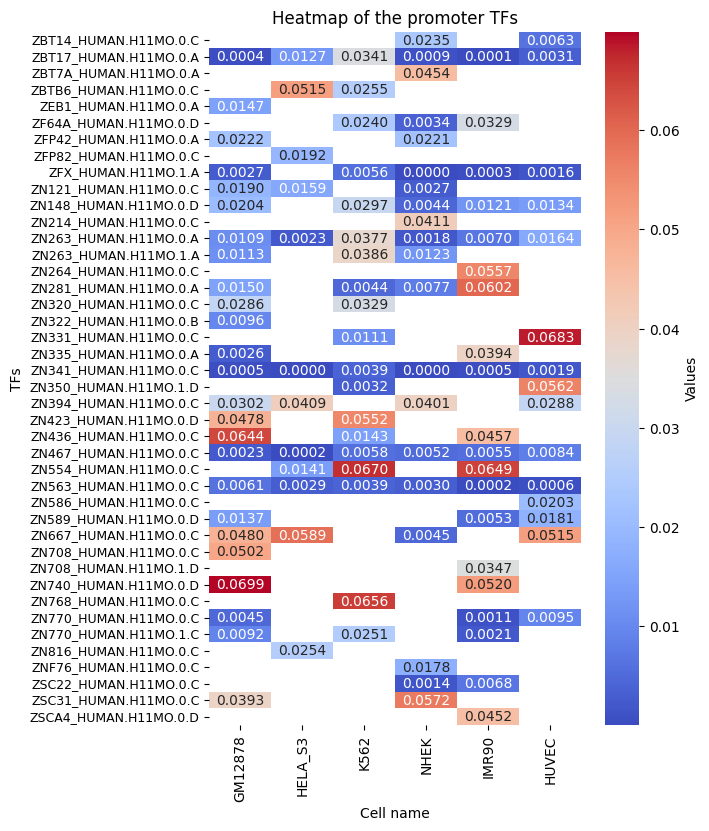

Supplement: vbaf221_Supplementary_Data [file vbaf221_supplementary_data.zip › Supplementary_file4/4-pro.png]
