# Supplementary material for: DeepEPI: CNN-transformer-based model for extracting TF interactions through predicting enhancer-promoter interactions
Source: Bioinform Adv. 2025 Sep 17;5(1):vbaf221. doi: 10.1093/bioadv/vbaf221 (PMC12478696; doi:10.1093/bioadv/vbaf221)
Supplement: vbaf221_Supplementary_Data [file vbaf221_supplementary_data.zip › supplementary_file6/Heatmap-Frequency.docx]

# TFs Frequency in Enhancers

**GM12878**

**HeLa**

**Huvec**

**IMR90**

**K562**

**NHEK**

# TFs Frequency in Promoters

**GM12878**

**HeLa**

**Huvec**

**IMR90**

**K562**

**NHEK**
