# Supplementary material for: DeepEPI: CNN-transformer-based model for extracting TF interactions through predicting enhancer-promoter interactions
Source: Bioinform Adv. 2025 Sep 17;5(1):vbaf221. doi: 10.1093/bioadv/vbaf221 (PMC12478696; doi:10.1093/bioadv/vbaf221)
Supplement: vbaf221_Supplementary_Data [file vbaf221_supplementary_data.zip › Supplementary_file9/guidance_for_output_files.docx]

**--- Overall Metrics --- With example of AR TF**

- **Total number of TF binding sites: 59**
  - **Explanation:** This is the absolute count of individual AR TF binding events identified in your dataset. Each row in your input .bed file represents one such binding event. This is the fundamental size of your discovery.
- **Total number of unique enhancer regions associated with TF binding: 45**
  - **Explanation:** This tells you how many distinct genomic regions you've identified as enhancers that have at least one AR TF binding site within them. Since 45 is less than 59 (total TF sites), it implies that some enhancer regions have more than one AR TF binding site within them. This is an important finding, as it suggests certain enhancers might be "hotspots" for AR binding.
- **Number of TF binding sites fully contained within their associated enhancer: 59 (100.00%)**
  - **Explanation:** This confirms a critical relationship. It means that every single AR TF binding site you've identified falls entirely within the boundaries of its designated associated enhancer region. This strengthens the biological relevance of these enhancer-TF associations, as it's expected for TFs to bind *within* regulatory elements.
- **Average distance from TF site center to associated enhancer center: 754.05 bp**
  - **Explanation:** This metric quantifies the average spatial relationship between the TF binding site and its enhancer. Since all TF sites are within enhancers, this isn't a "distance to nearest" metric. Instead, it measures how far, on average, the center of the smaller AR TF binding site is from the center of its larger enhancer region. An average of 754.05 bp suggests that the AR TF sites are generally not precisely in the dead center of their enhancers but can be offset, usually within a kilobase from the center.

**--- Chromosomal Distribution of TF Sites ---**

This section tells you how your identified AR TF binding sites are distributed across different chromosomes in the genome.

- **Explanation:** This is a count of how many AR TF binding sites were found on each specific chromosome.
  - chr1: 2 means 2 AR TF sites were found on chromosome 1.
  - chr11: 15 means 15 AR TF sites were found on chromosome 11.
  - ...and so on for all listed chromosomes.
- **Significance:** This distribution can highlight chromosomes that are particularly rich in AR regulatory activity, which might be biologically relevant (e.g., if target genes for AR are concentrated on certain chromosomes). Chromosome 11 and 17 appear to have a higher density of AR binding sites in your dataset.

**--- Number of TF Sites per Unique Enhancer Statistics ---**

These metrics quantify how many AR TF binding sites are typically found within a single enhancer region.

- **count: 45.000000**
  - **Explanation:** This count matches the number of unique enhancer regions.
- **mean: 1.311111**
  - **Explanation:** On average, each unique enhancer region contains about 1.31 AR TF binding sites. This confirms that while some enhancers have only one site, others must have multiple.
- **std: 0.763432**
  - **Explanation:** The standard deviation indicates the variability in the number of sites per enhancer. A standard deviation less than 1, while the mean is slightly above 1, suggests that most enhancers have one sites.
- **min: 1.000000**
  - **Explanation:** The minimum number of AR TF sites observed in any single enhancer is 1.
- **25% (1st Quartile): 1.000000**
  - **Explanation:** 25% of the unique enhancers contain 1 AR TF site.
- **50% (Median): 1.000000**
  - **Explanation:** 50% (half) of the unique enhancers contain exactly 1 AR TF site. This is a key finding: a majority of your identified enhancers are associated with a single AR binding event.
- **75% (3rd Quartile): 1.000000**
  - **Explanation:** 75% of the unique enhancers contain 1 AR TF site. This further emphasizes that enhancers with multiple sites are less common, as the 75th percentile is also 1.
- **max: 4.000000**
  - **Explanation:** The maximum number of AR TF sites found within any single enhancer is 4. This highlights that there are indeed "hotspot" enhancers with concentrated AR binding.
- **Significance:** This tells you about the density of AR binding within enhancers. The fact that the median and 25th/75th percentiles are 1 suggests that while multiple binding events occur, a single binding event per enhancer is the most common scenario.

**--- Relative Position of TF Site within Enhancer Statistics (0 = enhancer start, 1 = enhancer end) ---**

These metrics describe where the AR TF binding sites are located *within* their associated enhancer regions, normalized from the start (0) to the end (1) of the enhancer.

- **count: 59.000000**
  - **Explanation:** There are 59 relative position values, one for each TF site.
- **mean: 0.398359**
  - **Explanation:** On average, AR TF binding sites are located at about 39.8% of the way through the enhancer region, starting from the enhancer's beginning. This suggests a slight tendency for AR binding to occur in the **first half** of the enhancer, somewhat closer to the start than the middle.
- **std: 0.265910**
  - **Explanation:** The standard deviation shows a considerable spread in relative positions. This indicates that while there's a mean tendency, AR sites are not strictly confined to a very narrow region within enhancers; they can be found across a broad range of positions.
- **min: 0.022341**
  - **Explanation:** The closest an AR TF site's start is to the beginning of its enhancer is about 2.2% of the way through the enhancer.
- **25% (1st Quartile): 0.189063**
  - **Explanation:** 25% of the AR TF sites begin within the first ~19% of their enhancer's length.
- **50% (Median): 0.293098**
  - **Explanation:** Half of the AR TF sites begin within the first ~29.3% of their enhancer's length. This reinforces the idea that AR binding is generally found in the **earlier portions** of these enhancers.
- **75% (3rd Quartile): 0.622541**
  - **Explanation:** 75% of the AR TF sites begin within the first ~62.3% of their enhancer's length. This means only 25% of sites are found in the last ~37.7% of the enhancer.
- **max: 0.934978**
  - **Explanation:** The furthest an AR TF site's start is from the beginning of its enhancer is about 93.5% of the way through. This shows that while less common, AR sites can sometimes be found very close to the very end of an enhancer.
- **Significance:** This metric provides spatial insight. The tendency for AR binding to be in the earlier parts (0-0.5 range) of the enhancers might suggest functional implications or a preference for certain local sequence contexts.
