# Supplementary material for: DeepEPI: CNN-transformer-based model for extracting TF interactions through predicting enhancer-promoter interactions
Source: Bioinform Adv. 2025 Sep 17;5(1):vbaf221. doi: 10.1093/bioadv/vbaf221 (PMC12478696; doi:10.1093/bioadv/vbaf221)
Supplement: vbaf221_Supplementary_Data [file vbaf221_supplementary_data.zip › Supplementary_file1.pdf]

Position Weight Matrices

The extraction of Position Weight Matrices (PWMs) for motif matching from the attention layer is performed through several stages using a combination of attention weights, convolutional layers, and motif scanning. The following steps outline how PWMs are derived from our model and how it is used to study TF-TF interactions:

PWM Extraction Process and TF—TF interaction Analysis:

1. Model Training and Head Weights Capture: After training the model, we input positive test samples for each cell line into the trained model and capture the attention weights from the 16 heads. Each head is of size 246 x 246, where 148 parts correspond to enhancers and 98 parts correspond to promoters. Our goal is to know each 246 regions correspond to what motifs for the promoters and enhancers.
2. Feature Extraction via CNN and Max Pooling:
    - To identify motifs, we first apply CNN and max pooling on the positive test sequences. We use a sliding window with a kernel size of 40 to scan and extract features from the sequences. For example, if we have 129 positive samples, after scanning, we obtain two arrays with shapes (129, 1961) for promoters and (129, 2961) for enhancers.
    - We then extract the trained convolutional weights, resulting in arrays of shape (129, 1961, 128) for promoters and (129, 2961, 128) for enhancers, where 128 refers to the number of filters used in the CNN.
3. Max Pooling for Feature Importance:
    - To identify the most important features, we apply max pooling with a window size of 20 and a stride of 20 on the convolutional weights. This reduces the dimensionality to (129, 148, 128) for enhancers and (129, 98, 128) for promoters. By doing this, we can pinpoint which features correspond to which part of the sequence and convolutional filters, allowing us to map these features directly to the PWMs.
4. Motif Selection and PWM Construction:
    - Since we have 128 filters, each motif corresponds to 128 potential features. To reduce the noise and discard nonimportant features, we discard features associated with low filter weights, retaining only the most relevant ones. For each of the 246 positions, only the best-scoring motif remains. Once the motifs are identified, we aggregate nucleotide occurrences across these positions to construct Position Weight Matrices (PWMs), which represent nucleotide frequencies within the motifs.
5. TF Matching with TOMTOM:
    - The selected motifs are analyzed using the TOMTOM tool to match them against known transcription factors (TFs), using an E-value threshold of less than 0.07 to identify the best matching TFs for each motif. Once identified, the relevance of these motifs is further assessed by utilizing the multihead attention mechanism, which helps determine whether motifs interact based on their attention scores. In the multihead attention layer, each attention head learns to focus on different parts of the sequence, capturing diverse relationships between motifs in the enhancer and promoter regions. Each head produces an attention score matrix, where higher scores indicate stronger interactions between specific motifs. By analyzing these attention scores across multiple heads, we can determine which

motif pairs are more relevant to the model's understanding of the sequence. These high-scoring motifs are mapped to specific TFs through TOMTOM, allowing us to infer potential TF-TF interactions. To validate these predicted interactions, we use the BioGRID tool, which provides experimentally validated data on physical, genetic, and indirect TF interactions. This helps confirm whether the high-scoring motif pairs indeed correspond to real, biologically significant TF interactions, adding a layer of biological relevance to the model's predictions. The final list of motifs and their E-values, as well as detailed information on the TF-TF interactions across six cell lines, are provided in Supplementary file5. Additionally, the complete code with comments, outlining the steps for constructing PWMs, is also included in Colab link.
