# Supplementary material for: DeepEPI: CNN-transformer-based model for extracting TF interactions through predicting enhancer-promoter interactions
Source: Bioinform Adv. 2025 Sep 17;5(1):vbaf221. doi: 10.1093/bioadv/vbaf221 (PMC12478696; doi:10.1093/bioadv/vbaf221)
Supplement: vbaf221_Supplementary_Data [file vbaf221_supplementary_data.zip › Supplementary_file2.pdf]

In this supplementary material, we provide a link to the **WandB tool**, where we monitored the training phase of a single cell line (**KHEK**), including **GPU and CPU consumption over five epochs**. You can view the dynamic images through these links. Also in preprocessign phase took nearly 3 GB for both Embed-OneHot and DNA2Vec and took 9 GB for OneHot.

https://api.wandb.ai/links/sf-tabatabaeii73-sharif-university-of-technology/e53qfqmr

https://api.wandb.ai/links/sf-tabatabaeii73-sharif-university-of-technology/iti04dra

https://api.wandb.ai/links/sf-tabatabaeii73-sharif-university-of-technology/3cwnhu6a

https://api.wandb.ai/links/sf-tabatabaeii73-sharif-university-of-technology/j3oxqktp

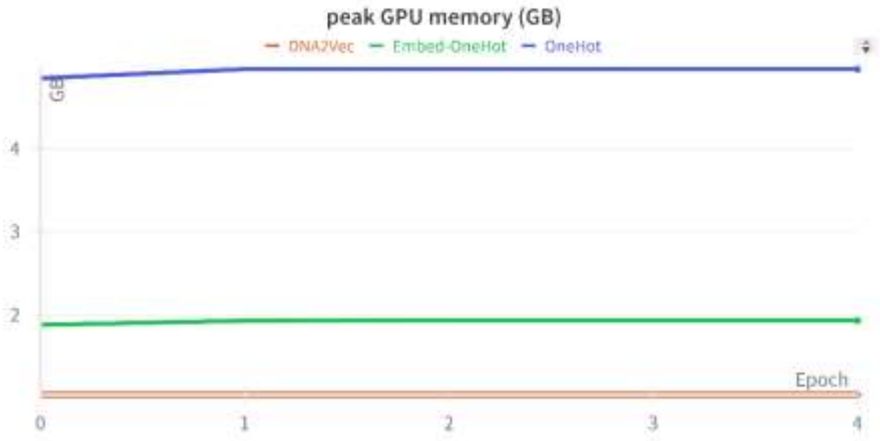

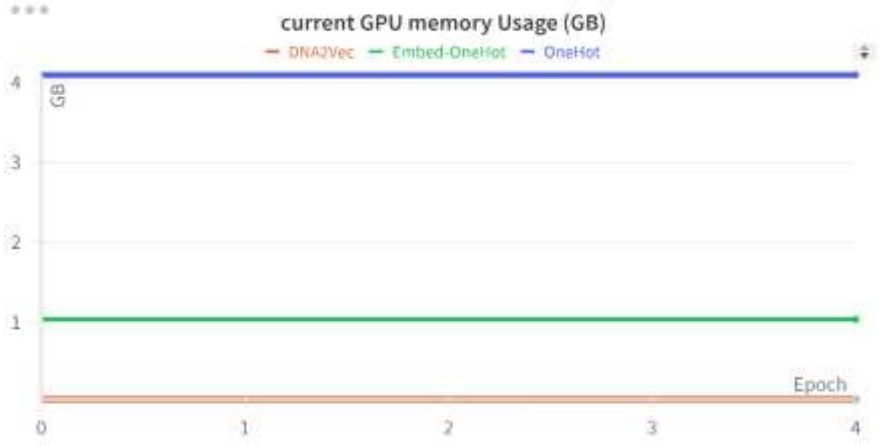

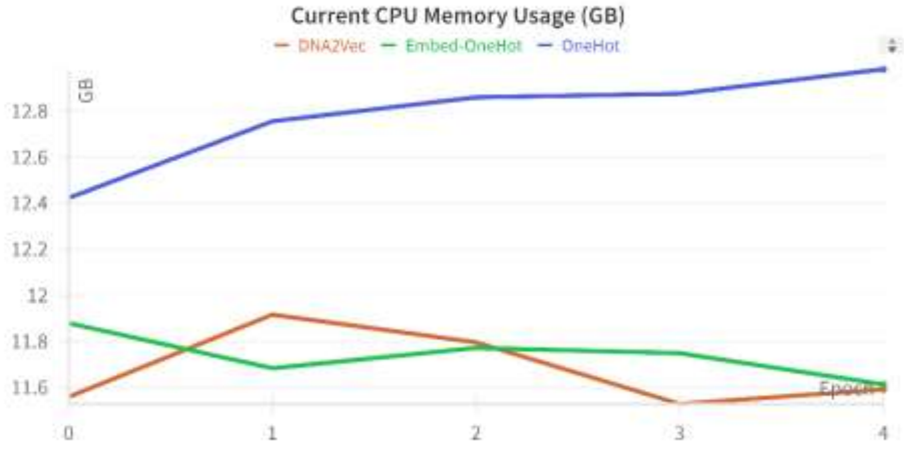
Current CPU Memory Usage (GB)
DNA2Vec
Embed-OneHot
OneHot
GB
12.8
12.6
12.4
12.2
12
11.8
11.6
Epoch
0
1
2
3
4

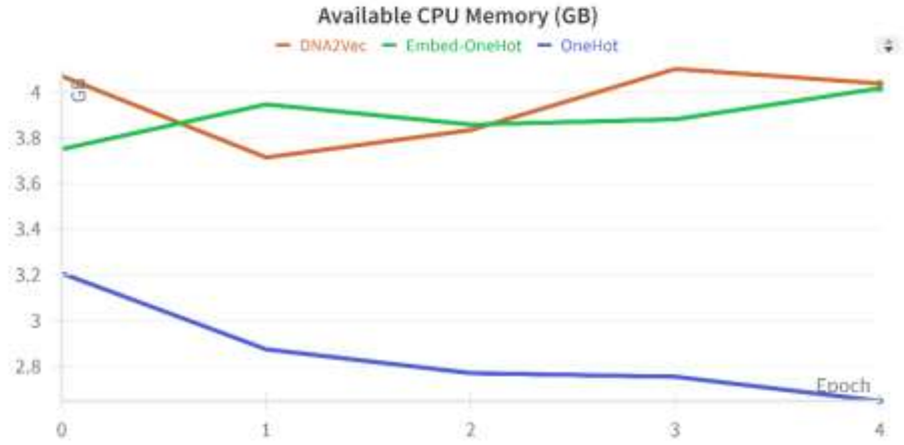
Available CPU Memory (GB)
DNA2Vec
Embed-OneHot
OneHot
4
3.8
3.6
3.4
3.2
3
2.8
Epoch
0
1
2
3
4
