# Supplementary material for: DeepEPI: CNN-transformer-based model for extracting TF interactions through predicting enhancer-promoter interactions
Source: Bioinform Adv. 2025 Sep 17;5(1):vbaf221. doi: 10.1093/bioadv/vbaf221 (PMC12478696; doi:10.1093/bioadv/vbaf221)
Supplement: vbaf221_Supplementary_Data [file vbaf221_supplementary_data.zip › Supplementary_file7.pdf]

Data Sources:

The interactions analyzed in this study were derived from BioGRID (Biological General Repository for Interaction Datasets), a comprehensive database that integrates interaction data from both high-throughput experiments and smaller-scale studies. BioGRID curates a wide array of physical and genetic interactions from primary literature and large-scale screens, offering a robust resource for the validation of molecular interactions.

Interaction Extraction Process from BioGRID:

Our method for identifying transcription factor (TF) interactions begins with the extraction of motifs from a deep learning model using multi-head attention layers. We analyzed motif interactions at specific positional heads, focusing on those with the highest attention scores (5 to 8 positions), based on the model's learned attention weights. These motifs were then matched to significant transcription factors using the TOMTOM tool, retaining only those with an E-value lower than 0.07, ensuring statistical significance.

Subsequently, we validated the identified TF-TF interactions by querying the BioGRID database to classify and confirm the nature of each interaction. This multi-step approach allowed us to rigorously validate our results and ensure their accuracy.

BioGRID's data was crucial in validating the following types of interactions, categorized as:

- **Physical Interactions (PI):** Direct protein-protein binding**.**
- **Genetic Interactions (GI):** How one gene's mutation or activity influences another gene's function or expression.
- **Indirect Interactions (II):** Interactions mediated by intermediate steps, such as regulatory pathways or signaling cascades.

Additionally, interactions were classified by:

- **Throughput (LT vs. HT):** Denoting whether the interaction data was derived from low-throughput **(LT)** experiments, which are smaller and more focused, or from high-throughput **(HT)** studies, which involve large-scale screens.
- **Non-redundancy (NR):** Interactions designated as non-redundant (NR) are unique and represent distinct regulatory relationships without duplication.

This thorough methodology, combining motif extraction, statistical analysis, and BioGRID validation, ensured a robust and credible presentation of TF-TF interactions.

Key Metrics in table result:

- **M# Enhancer and M# Promoter:** Represent motif numbers learned by heads of multi-head attention. These motifs are not explicitly biological sequences but are learned features that correlate with biological enhancer and promoter regions. By visualizing the model's learned attention weights, we identified how these motifs interact, which allowed for the discovery of transcription factors (TFs) that potentially bind to these motifs.

1. **Physical Interaction (PI)**: This involves the direct binding between two biomolecules (e.g., SOX2 and CTCF). For example, in the NHEK cell line, the SOX2—CTCF interaction in the M-127

enhancer and M-72 promoter region is a physical interaction identified as PI, NR, LT:6, meaning it is non-redundant and detected using low-throughput methods six times.

2. **Indirect Interaction (II)**: These interactions are not due to direct binding but are mediated through intermediate regulatory networks. For instance, the PURA—EGR1 interaction in M-26 Enhancer and M-44 Promoter in NHEK is mediated through TOE1, showcasing an indirect interaction.
3. **Genetic Interaction (GI)**: Genetic interactions describe how the presence or activity of one gene influences the function of another. In GM12878, the SP4—SP1 interaction (GI, NR, LT:1) shows how these transcription factors influence each other's activity indirectly, affecting phenotype.

Physical interaction:PI(Direct binding between biomolecules).<br>Indirect_Interaction=II(Mediated through intermediate steps or regulatory networks, not direct binding.).<br>Genetic Interaction=GI(How genes influence each other's effects on phenotypes).<br>Non-redundant=NR, Low Throughput=LT, High Throughput=LT,

Motif#	NHEK	Source	Descryption
M-20 Enhancer — M-51 promoter	YY1—SP1(PI, NR, LT: 6), YY1—SP3(PI, NR, LT:1),YY1—KLF16(PI, NR, HT: 1), YY1—THAP11(PI, NR, HT: 1),YY1—YY1(PI, NR, LT: 3),TBX2—EGR1(PI, NR, LT: 2),RARG—RXRA(PI, NR, LT: 1),	BioGRID	
M-26 Enhancer—M-44 promoter	PURA—SP1(PI, NR, LT: 1), PURA—PURA(PI, NR, LT: 1), PURA – EGR1(II), RXRA—SP1(PI, NR, LT: 2), RXRA—RXRA(PI, NR, LT: 2),	BioGRID	PURA – EGR1 are connected through TOE1.
M-52 Enhancer—M-65 promoter	RXRA—SP1(PI, NR, LT: 2), RXRA—NR1H4(PI, NR, LT: 4), RXRA—RXRA(PI, NR, LT: 2), RARG—RXRA(PI, NR, LT: 1),	BioGRID	
M-56 Enhancer—M-52 promoter	NR1H3—RXRA (PI, NR, HT: 1)(PI, NR, LT: 3)	BioGRID	
M-68 Enhancer—M-65 promoter	LEF1—E2F1(PI, NR, LT: 1)	BioGRID	
M-75 Enhancer—M-38promoter	STAT3—RXRA(PI, NR, HT: 1), STAT1—RXRA(PI, NR, HT: 1)(PI, NR, LT: 1)<br>, STAT3—HESX1(PI, NR, HT: 1),,STAT3—KLF15(PI, NR, HT: 1)	BioGRID	
M-127 Enhancer—M-72 promoter	SOX2—SP2(PI, NR, HT: 1), SOX2—PATZ1(PI, NR, HT: 1), SOX2—CTCF(PI, NR, HT: 1), ZBTB17—ZBTB17(PI, NR, LT: 2), ZBTB17—E2F6(PI, NR, LT: 1)	BioGRID	
M-15 Enhancer—M-50 promoter	CTCF—YY1(PI, NR, LT: 1)	BioGRID	

Motif#	GM12878	Source	Descrtption
M-3 Enhancer—M-49 Promoter	VEZF1—SP4(PI, NR, HT: 1), KLF6—SP1(PI, NR, LT: 5), RXRA—CTCF(PI, NR, LT: 1), RXRA—SP1(PI, NR, LT: 2), RXRA—TAF1B(PI, NR, LT: 1), RXRA—RXRA(PI, NR, LT: 2),	BioGRID	
M-13 Enhancer—M-63 Promoter	KLF6—SP1(PI, NR, LT: 5), KLF6—KLF15,MAZ—SP1, MAZ--SP2, MAZ—E2F4,MAZ—E2F6, MAZ—EGR1, ZNF263—SP1, ZNF263—SP2, ZNF263—MAZ, ZNF263—E2F6, ZNF263—EGR1, ZNF263—E2F4, ZNF467—PATZ1(PI, NR, HT:2), ZNF467—SP1, ZNF467—EGR1(II), WT1—THAP1(PI, NR, HT:1), KLF15—SP4, KLF15—SP2(PI, NR, HT:1), KLF15—KLF6, KLF15—SP3, KLF15—MAZ(PI, NR, HT:2), PATZ1—PATZ1(PI, NR, LT:1), VEZF1—SP4(PI, NR, HT:1), KLF6—SP1(PI, NR, LT:5), RARA—RXRA(PI, NR, HT: 2)(PI, NR, LT: 18), RARA—RXRB, RARA—RARG(PI, NR, HT: 2), RARA—RARB(PI, NR, HT: 2)(PI, NR, LT: 1), RARA—SP1(PI, NR, LT: 4), RARA—RARA(PI, NR, LT: 4),	Both BioGRID & Literature	ZNF467—EGR1(II), are connected through TOE1
M-57 Enhancer—M-63 Promoter	RXRA—RARA(PI, NR, HT: 2) (PI, NR, LT: 18), RXRA—RARG(PI, NR, LT: 1), RXRA—SP1(PI, NR, LT: 2), RXRA—RARB(PI, NR, LT: 2),	BioGRID	
M-57 Enhancer—M-91 Promoter	TLX1—TLX1(PI, NR, LT: 1), TLX1—ZNF281(PI, NR, HT: 1), TLX1—MNX1(PI, NR, HT: 1),	BioGRID	
M-103 Enhancer—M-49 Promoter	WT1—WT1(PI, NR, HT:1), WT1—THAP1(PI, NR, HT:1), WT1—EGR1(PI, NR, LT:1), SP4—SP4(PI, NR, HT: 1), SP4—SP1(GI, NR, LT: 1), SP4—SP3(GI, NR, LT: 1), SNAI1—EGR1(PI, NR, LT: 2), SNAI1—PURA(PI, NR, HT: 1), MNX1—TLX1(PI, NR, HT: 1),	BioGRID	
M-113 Enhancer—M-63 Promoter	PURA—SP1(PI, NR, LT: 1), PURA—EGR1(II),	BioGRID	
M-121 Enhancer—M-63 Promoter	GCR—SP1(PI, NR, HT: 1), GCR—ZFX(PI, NR, LT: 1), RXRG—RARB(PI, NR, HT: 3)(PI, NR, LT: 1), RXRG—RARG (PI, NR, LT: 1) (PI, NR, HT: 1), RXRG—RXRA(PI, NR, HT: 3)	BioGRID	
M-122 Enhancer—M-42 Promoter	MEIS3—VEZF1(PI, NR, HT:1), MEIS2—SP1(PI, NR, LT: 1), ZNF324A—ZNF467(PI, NR, HT: 1),	BioGRID	
M-122 Enhancer—M-95 Promoter	MEIS2—TLX1(PI, NR, LT: 1),	BioGRID	

Motif#	K562	Source
M-38 Enhancer—M-54 Promoter	MNX1—TLX1(PI, NR, HT: 1), FOXO1—SP1(II), FOXO1—SP2(II), FOXO1—SP3(II), SP4—SP1(GI, NR, LT: 1), SP4—SP4(PI, NR, HT: 1), SP4—VEZF1(PI, NR, HT:1), SP4—SP3(GI, NR, LT: 1), KLF3—ZNF148(PI, NR, HT: 1), MAZ—SP1(II), MAZ—SP2(II), MAZ—ZNF263(II), MAZ—EGR1(II),	Both BioGRID & Literature[2], [3], [4], [11]
M-110 Enhancer—M-54 Promoter	TLX1—TLX1(PI, NR, LT: 1), MZF1—SP3(II), MZF1—SP2(II), SMAD2—SP1(PI, NR, LT: 5), BCL11A—EGR1(II), BCL11A—SP1(PI, NR, HT:1),	Both BioGRID & Literature[2], [12]
M-146 Enhancer—M-54 Promoter	RXRA—SP1(PI, NR, LT: 2), GATA1—SP1(PI, NR, LT: 1), GATA1—SP2(II), GATA1—EGR1(II),	Both BioGRID & Literature[13], [14]
M-96 Enhancer—M-1 Promoter	RXRA—RXRA(PI, NR, LT: 2), SP2—MAZ (II), VEZF1—SP4(PI, NR, HT:1),	Both BioGRID & Literature[2]
M-110 Enhancer—M-3 Promoter	SMAD2—TAF1(PI, NR, HT: 1),	BioGRID
M-122 Enhancer—M-1 Promoter	PATZ1—PATZ1(PI, NR, LT:1),	BioGRID
M-122 Enhancer—M-69 Promoter	TAF1—KLF16(PI, NR, HT: 1), RXRA—RARG(PI, NR, LT: 1), PATZ1—RXRA, SP2—SP1(PI, NR, HT: 1),	Both BioGRID & Literature [2]
M-127 Enhancer—M-33 Promoter	ZNF467—SP1(II), ZNF467—PATZ1(PI, NR, HT: 2), ZNF467—ZNF770(PI, NR, HT: 2)	BioGRID

Motif#	HUVEC	Source
M-6 Enhancer—M-46 Promoter	BPTF—MAZ(PI, NR, LT:2)(PI, NR, HT:1), NFATC1—EGR1(PI, NR, LT:1), NFATC1—EGR2(PI, NR, LT:1), NFIC—KLF3(PI, NR, HT:1)(PI, NR, LT:1), NFIC—RFX1(PI, NR, LT:1),	BioGRID
M-26 Enhancer—M-46 Promoter	P73—SP1(PI), ZNF467—PATZ1(PI, NR, HT: 2), ZNF467—EGR1(II), RARG—RXRA(PI, NR, LT: 1), RXRA—RXRA(PI, NR, LT: 2), BCL11A—EGR1(II)	Both BioGRID & Literature [1], [2]
M-46 Enhancer—M-46 Promoter	ISL1—MAFA(PI, NR, LT:1), WT1--EGR1(PI, NR, LT:1), WT1—WT1(PI, NR, HT:1), WT1—THAP1(PI, NR, HT:1), ZNF263—PURA(PI, NR, HT: 1), ZNF263—EGR1(II), ZNF263—SP2(II), ZNF263—MAZ(II),	Both BioGRID & Literature [3]
M-41 Enhancer—M-87 Promoter	CEBPZ—ZNF467(PI, NR, HT: 2), BCL11A—SP1(PI, NR, HT:1), STAT1—RXRA(PI, NR, HT: 1)(PI, NR, LT: 1) , RXRA—CLOCK(PI, NR, LT:2), RXRA—RXRA(PI, NR, LT: 2), RXRA—SP1(PI, NR, LT: 2),	BioGRID
M-44 Enhancer—M-5 Promoter	RXRA—KLF5(PI, NR, LT: 2), MAZ—KLF15(PI, NR, HT:2), MAZ—KLF5(PI, NR, HT:1), MAZ—EGR1(II), MAZ—SP1(II),MAZ—SP2(II), TBX21—SP1(PI, NR, HT:1), SP2—SP1(PI, NR, HT:1), SP2—MAZ(II), SP2—EGR1(II), SP2—KLF15(PI, NR, HT:1), SP3—SP1(PI, NR, LT:8), SP3—SP4(GI, NR, LT:1), SP3—KLF15(II),	Both BioGRID & Literature [2], [3], [4]
M-61 Enhancer—M-31 Promoter	NR1H3--RXRA(PI, NR, HT: 1),(PI, NR, LT: 3)	BioGRID

Motif#	HELA-S3	Source
M-124 Enhancer—M-57 Promoter	MAZ--SP2(II), MAZ--SP1(II), SMAD4--RFX1(PI, NR, HT: 1), EGR1—MAZ(II), PAX5—MAZ(II), PAX5--SP1(II), KLF15--SP4(II), KLF15--SP2(PI, NR, HT:1), KLF15--SP3(II), KLF15--KLF6(II), SMAD4--LEF1(PI, NR, LT: 1), SMAD4--SP1(PI, NR, LT: 5)	Both BioGRID & Literature [2], [3], [4], [5], [6]
M-76 Enhancer—M-57 Promoter	TAL1--SP1(II), TAL1--SP2(II), GATA2--SP1(II), GATA2--SP2(II), GATA1--SP1(PI, NR, LT: 1), GATA1--SP2(II), SP1--SP3(PI, NR, LT: 8), SP1--SP1(PI, NR, LT: 1), SP1--SP2(PI, NR, HT: 1)	Both BioGRID & Literature [2], [7], [8]
M-48 Enhancer—M-36 Promoter	IRF1--SP1(II), IRF1--KLF15(II), IRF1--EGR1(II), IRF1--SP2(PI, NR, LT: 1), IRF1--E2F4(II), RXRA--SP1(PI, NR, LT: 2), RXRA--RXRA(PI, NR, LT: 2), NRF1--KLF15(PI, NR, HT:1), ZNF770—ZNF467(PI, NR, HT: 2), STAT1—RXRA(PI, NR, HT: 1)(PI, NR, LT: 1) , IRF2—NFKB1(PI, NR, LT: 1),	Both BioGRID & Literature [2], [9], [10]
M-62 Enhancer—M-62 Promoter	NR1H3--RXRA(PI, NR, HT: 1) (PI, NR, LT: 3), PATZ1—PATZ1(PI, NR, LT:1)	BioGRID
M-77 Enhancer—M-72 Promoter	SOX2--SP2(PI, NR, HT: 1), SOX2--PATZ1(PI, NR, HT:1), SOX2--GATA4(PI, NR, HT: 2), FOS--MAZ(PI, NR, LT:2), JUN--KLF5(PI, NR, LT: 1), JUN--MAZ(PI, NR, LT:2), JUN--SP2(II), JUN--SP1(PI, NR, LT: 6), BACH1—NR1D1(PI, NR, HT: 1), BACH1—PATZ1(PI, NR, LT:1), BACH1--SP2(II), BACH2--PATZ1(PI, NR, LT:1) (PI, NR, HT:1), BACH2--ZBTB17(PI, NR, HT: 1), JUND--SP2(II), NFE2L2--GATA1(PI, NR, LT: 2), PATZ1--GATA4(PI, NR, HT:1), PATZ1--KLF1(PI, NR, HT:1), RXRA--KLF5(PI, NR, LT: 2), THAP1--THAP1(PI, NR, HT:5), PATZ1—PRDM14(PI, NR, HT:1),	Both BioGRID & Literature [2]
M-54 Enhancer—M-39 Promoter	CTCF—YY1(PI, NR, LT: 1)	BioGRID

Motif#	IMR90	Source
M-28 Enhancer—M-40 Promoter	THAP1—THAP1(PI, NR, HT:5), THAP1—KLF12(PI, NR, HT:1), THAP1—WT1(PI, NR, HT:1), RXRA—NR1H4(PI, NR, LT: 4), RXRA—SP1(PI, NR, LT: 2),	BioGRID
M-56 Enhancer—M-9 Promoter	MNX1—TLX1(PI, NR, HT: 1), BCL11A—SP1(PI, NR, HT:1),	BioGRID
M-61 Enhancer—M-93 Promoter	VEZF1—SP4(PI, NR, HT:1), RXRA—RXRA(PI, NR, LT: 2),, PATZ1—PATZ1(PI, NR, LT:1),	BioGRID
M-68 Enhancer—M-42 Promoter	RARG—RXRA(PI, NR, LT: 1), KLF6—SP1(PI, NR, LT:5), SMAD3—PLAG1(PI, NR, HT:1), MAZ—EGR1(II), MAZ—SP1(II), MAZ—SP2(II), MAZ—KLF15(PI, NR, HT:2), NFIC—KLF3(PI, NR, HT:1) (PI, NR, LT:1), NFIC—KLF16(PI, NR, LT:1), NFIC—TLX1(PI, NR, HT: 1), NFIC—ZNF148(PI, NR, HT:1), SP3—SP1(PI, NR, LT:8), SP3—SP4(GI, NR, LT:1), SP3—KLF15(II), TLX1—TLX1(PI, NR, LT: 1), NR4A2—RXRA(PI, NR, LT: 3), ZNF490—ZNF770(PI, NR, HT:1), SMAD3—SP1(PI, NR, HT:1) (PI, NR, LT:8), SMAD3—TAF1(PI, NR, HT:1),	Both BioGRID & Literature [3], [4], [6]

M-107 Enhancer—M-87 Promoter	CLOCK—RXRA(PI, NR, LT: 2), RXRA—PPARG(PI, NR, HT: 3)(PI, NR, LT: 36), RXRA—PPARA(PI, NR, LT: 6), RARA—RXRA(PI, NR, HT: 2) (PI, NR, LT: 18), RARA—SP1(PI, NR, LT: 4), RARA—PPARG(PI, NR, LT: 1)(PI, NR, HT: 1),	BioGRID
M-128 Enhancer—M-51 Promoter	ZNF324A—ZNF467(PI, NR, HT: 1), NR1H3—RXRA(PI, NR, HT: 1) (PI, NR, LT: 3),	BioGRID

[1] G. Koutsodontis, E. Vasilaki, W. C. Chou, P. Papakosta, and D. Kardassis, "Physical and functional interactions between members of the tumour suppressor p53 and the Sp families of transcription factors: Importance for the regulation of genes involved in cell-cycle arrest and apoptosis," *Biochemical Journal*, vol. 389, no. 2, 2005, doi: 10.1042/BJ20041980.

[2] W. Mao, D. Kostka, and M. Chikina, "Modeling Enhancer-Promoter Interactions with Attention-Based Neural Networks," 2017, doi: 10.1101/219667.

[3] F. Ullah, S. Jabeen, M. Salton, A. S. N. Reddy, and A. Ben-Hur, "Evidence for the role of transcription factors in the co-transcriptional regulation of intron retention," *Genome Biol*, vol. 24, no. 1, 2023, doi: 10.1186/s13059-023-02885-1.

[4] J. Song *et al.*, "Transcriptional regulation by zinc-finger proteins Sp1 and MAZ involves interactions with the same cis-elements.," 2003. doi: 10.3892/ijmm.11.5.547.

[5] Y. Liu, T. Cheng, Y. Du, X. Hu, and W. Xia, "LncRNA LUCAT1/miR-181a-5p axis promotes proliferation and invasion of breast cancer via targeting KLF6 and KLF15," *BMC Mol Cell Biol*, vol. 21, no. 1, 2020, doi: 10.1186/s12860-020-00310-0.

[6] J. Li, Y. Yang, B. Jiang, X. Zhang, Y. Zou, and Y. Gong, "Sp1 and KLF15 regulate basal transcription of the human LRP5 gene," *BMC Genet*, vol. 11, 2010, doi: 10.1186/1471-2156-11-12.

[7] E. Ravet *et al.*, "Characterization of DNA-binding-dependent and -independent functions of SCL/TAL1 during human erythropoiesis," *Blood*, vol. 103, no. 9, 2004, doi: 10.1182/blood-2003-05-1689.

[8] G. S. Ivanov *et al.*, "Sp and GATA factors are critical for Apolipoprotein AI downstream enhancer activity in human HepG2 cells," *Gene*, vol. 323, no. 1–2, 2003, doi: 10.1016/j.gene.2003.08.014.

[9] D. P. Wang *et al.*, "Apolipoprotein L1 is transcriptionally regulated by SP1, IRF1 and IRF2 in hepatoma cells," *FEBS Lett*, vol. 594, no. 19, 2020, doi: 10.1002/1873-3468.13887.

[10] Y. Chinenov, M. Coppo, R. Gupte, M. A. Sacta, and I. Rogatsky, "Glucocorticoid receptor coordinates transcription factor-dominated regulatory network in macrophages," *BMC Genomics*, vol. 15, no. 1, 2014, doi: 10.1186/1471-2164-15-656.

[11] C. Y. Wang, C. C. Yang, L. Der Hsiao, and C. M. Yang, “Involvement of FoxO1, Sp1, and Nrf2 in Upregulation of Negative Regulator of ROS by 15d-PGJ2 Attenuates H2O2-Induced IL-6 Expression in Rat Brain Astrocytes,” *Neurotox Res*, vol. 40, no. 1, 2022, doi: 10.1007/s12640-020-00318-6.

[12] S. Le Mée, O. Fromigué, and P. J. Marie, “Sp1/Sp3 and the myeloid zinc finger gene MZF1 regulate the human N-cadherin promoter in osteoblasts,” *Exp Cell Res*, vol. 302, no. 1, 2005, doi: 10.1016/j.yexcr.2004.08.028.

[13] A. M. M. Gannon and B. T. Kinsella, “Regulation of the human thromboxane A2 receptor gene by Sp1, Egr1, NF-E2, GATA-1, and Ets-1 in megakaryocytes,” *J Lipid Res*, vol. 49, no. 12, 2008, doi: 10.1194/jlr.M800256-JLR200.

[14] M. Flesch, “On the trail of cardiac specific transcription factors,” 2001. doi: 10.1016/S0008-6363(01)00218-8.
