# Supplementary material for: DeepEPI: CNN-transformer-based model for extracting TF interactions through predicting enhancer-promoter interactions
Source: Bioinform Adv. 2025 Sep 17;5(1):vbaf221. doi: 10.1093/bioadv/vbaf221 (PMC12478696; doi:10.1093/bioadv/vbaf221)
Supplement: vbaf221_Supplementary_Data [file vbaf221_supplementary_data.zip › Supplementary_file8.pdf]

CTCF—CTCF Interaction

The results indicate that we explored the presence of the CTCF-CTCF interaction across different cell lines using attention head weights and e-values to validate the motif interactions. Here's an interpretation of these findings:

General result:

CTCF-CTCF Interaction Across Cell Lines: In 4 cell lines, we found CTCF motifs in both enhancers and promoters (NHEK, K562, IMR90, HeLa. However, in GM12878 and HUVEC, CTCF motifs were only identified in promoters, with no enhancer CTCF motifs detected. And The E-values we provided indicate statistically significant matches for the CTCF motifs. Low E-values (<0.07) suggest that the CTCF motifs found are meaningful and not random. For example, this is one of our TF findings in the K562 cell with E-value=1.06984e-05.

Motif name	p-value	E-value	q-value
CTCF_HUMAN.H11MO.0.A	2.66794e-08	1.06984e-05	2.13969e-05

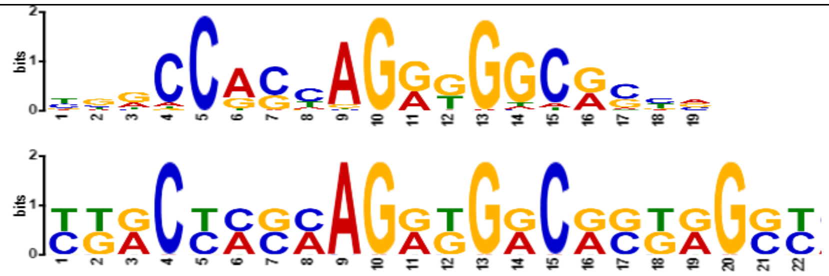

Cell Line-Specific results:

1. **NHEK Cell Line**:

   We identified CTCF motifs with low e-values in both enhancer and promoter regions, suggesting the potential for CTCF-CTCF interactions. The maximum weight value in the heads is 0.01335904, with some CTCF interaction weights, such as 0.011, being close to this highest value.

enhancer motifs number	,	promoter motifs number	Weights Heads	
110	--	50	0.0058708894	
110	--	72	0.008169798	**
15	--	50	0.008925457	**
15	--	72	0.0073384233	
69	--	50	0.011323608	***
69	--	72	0.0049459175	

promoter_50=> E-values:[0.0629389], promoter_72=>E-values:[0.0556589]

----

enhancer_110=>E-values:[0.00542415], enhancer_15=>E-values:[0.0213681], enhancer_69=>E-values:[0.00333733]

2. **K562 Cell Line**:

Multiple CTCF motifs were found in both enhancer and promoter regions with low e-values, supporting the presence of CTCF-CTCF interactions. The maximum weight value in the heads is 0.009835904, with some CTCF interaction weights, such as 0.0083, being close to this highest value.

enhancer motifs number	,	promoter motifs number	Weight Heads	
128	--	62	0.0051802583	
128	--	86	0.003456808	
33	--	62	0.003980127	
33	--	86	0.0041156695	
63	--	62	0.004457233	
63	--	86	0.008308462	**
85	--	62	0.005089614	
85	--	86	0.0036798532	
37	--	62	0.0043770196	
37	--	86	0.006328245	**

enhancers_128=>E-values:[1.06984e-05], enhancers_33=>E-values:[0.00498996], enhancers_63=>E-values:[0.000739451], enhancers_37=>E-values:[0.07139], enhancers_85=>E-values:[0.0455532]

----

promoter_62=>E-values:[0.0676863], promoter_86=>E-values:[0.0204654]

3. **IMR90 Cell Line**:

CTCF motifs were identified in both enhancer and promoter regions, suggesting robust CTCF-CTCF interactions in IMR90 cells. The maximum weight value in the heads is 0.011979799, with some CTCF interaction weights, such as 0.010, being close to this highest value.

enhancer motifs number	,	promoter motifs number	Weight Heads	
30	--	21	0.0042813374	
30	--	26	0.005628233	
30	--	34	0.004749145	
30	--	39	0.007897747	
30	--	47	0.010007866	***
104	--	21	0.0072735203	
104	--	26	0.0042464705	
104	--	34	0.0035996698	
104	--	39	0.009616403	**
104	--	47	0.008174326	**
86	--	21	0.0039910087	
86	--	26	0.004860879	
86	--	34	0.0042993184	
86	--	39	0.0033537492	
86	--	47	0.009340746	**

enhancers_104=>E-values:[0.0398185], enhancers_30=>E-values:[0.00542415], enhancers_86=>E-values:[0.00628993]

----

promoter_21=>E-values:[0.0685303], promoter_26=>E-values:[0.0501592], promoter_34=>E-values:[0.0286684], promoter_39=>E-values:[0.00609638], promoter_47=>E-values:[0.0611499]

4. **HeLa Cell Line**:

CTCF motifs were detected in both enhancer and promoter regions, with some significant attention head weights reaching up to 0.009802878. This suggests a potential for CTCF-CTCF interactions in HeLa cells. The maximum head weight value is 0.010914041.

enhancer motifs number	,	promoter motifs number	Weight Heads
116	--	26	0.003731239
116	--	51	0.00801879 **
116	--	65	0.0087749725
116	--	68	0.009802878 **
54	--	26	0.0033328992
54	--	51	0.0033499117
54	--	65	0.008876612 **
54	--	68	0.0048602214
enhancers_116 =>E-values:[0.0217952], enhancers_54=>E-values:[0.00410927]<br>--<br>promoters_26=>E-values:[0.0212079],promoters_51=>E-values:[0.0468433],<br>promoters_65=>E-values:[0.0670991],promoters_68=>E-values:[0.0625214]			

5. **GM12878 and HUVEC Cell Lines**:

In both GM12878 and HUVEC, CTCF motifs were detected only in the promoter regions, and no enhancer motifs were found. The absence of enhancer CTCF motifs suggests that CTCF-CTCF interactions might not occur, or are weaker, in these two cell lines.

For GM12878: promoters_87=>E-values:[0.0401842], promoters_14=>E-values:[0.0645904]<br>promoters_3=>E-values:[0.0499537]
For HUVEC : promoters_18=> E-values:[0.0365755]
