# Supplementary material for: DeepEPI: CNN-transformer-based model for extracting TF interactions through predicting enhancer-promoter interactions
Source: Bioinform Adv. 2025 Sep 17;5(1):vbaf221. doi: 10.1093/bioadv/vbaf221 (PMC12478696; doi:10.1093/bioadv/vbaf221)
Supplement: vbaf221_Supplementary_Data [file vbaf221_supplementary_data.zip › supplementary-file2.docx]

In this supplementary material, we provide a link to the **WandB tool**, where we monitored the training phase of a single cell line (**KHEK**), including **GPU and CPU consumption over five epochs**. You can view the dynamic images through these links. Also in preprocessign phase took nearly 3 GB for both Embed-OneHot and DNA2Vec and took 9 GB for OneHot.

<https://api.wandb.ai/links/sf-tabatabaeii73-sharif-university-of-technology/e53qfqmr>

<https://api.wandb.ai/links/sf-tabatabaeii73-sharif-university-of-technology/iti04dra>

<https://api.wandb.ai/links/sf-tabatabaeii73-sharif-university-of-technology/3cwnhu6a>

<https://api.wandb.ai/links/sf-tabatabaeii73-sharif-university-of-technology/j3oxqktp>
